# Altered cytoskeletal integrity underlies impaired platelet shape change and defective thrombus formation in ETV6‐related thrombocytopenia

**DOI:** 10.1111/bjh.70495

**Published:** 2026-04-28

**Authors:** Ivan P. Tesakov, Julia‐Jessica D. Korobkin, Daria V. Fedorova, Sofia V. Galkina, Maria M. Golomysova, Sergei I. Obydennyi, Anastasia A. Ignatova, Ekaterina‐Iva A. Adamanskaya, Eugenia V. Yushkova, Anna V. Pavlova, Pavel A. Zharkov, Igor I. Kireev, Galina A. Novichkova, Viktor G. Zgoda, Mikhail A. Panteleev, Anastasia N. Sveshnikova

**Affiliations:** ^1^ Department of Oncology, Hematology, Clinical Immunology, and Rheumatology University Hospital Tuebingen Tuebingen Germany; ^2^ Dmitry Rogachev National Medical Research Center of Pediatric Hematology, Oncology and Immunology Moscow Russia; ^3^ Center for Theoretical Problems of Physico‐Chemical Pharmacology Russian Academy of Sciences Moscow Russia; ^4^ A.N. Belozersky Institute of Physico‐Chemical Biology Lomonosov Moscow State University Moscow Russia; ^5^ Department of Proteomic Research and Mass Spectrometry V.N. Orekhovich Institute of Biomedical Chemistry Moscow Russia; ^6^ Faculty of Physics Lomonosov Moscow State University Moscow Russia

**Keywords:** bone marrow failure, ETV6, ETV6‐related thrombocytopenia, inherited platelet disorders, platelet function assays, platelet proteomics, thromboinflammation

## Abstract

ETV6‐related thrombocytopenia (ETV6‐RT) is an inherited platelet disorder caused by germline ETV6 variants. Despite recent progress, the mechanisms underlying platelet dysfunction in ETV6‐RT remain unclear. We investigated 12 patients from six families using functional assays, electron microscopy, quantitative proteomics and cytoskeletal imaging. Most patients exhibited mild‐to‐moderate thrombocytopenia with variable paediatric bleeding symptoms (median International Society on Thrombosis and Haemostasis Bleeding Assessment Tool 3, range 1–9) but consistently mild bleeding in adulthood (median 0, range 0–1). Ex vivo thrombus formation was reduced independent of platelet count. Electron microscopy revealed defective platelet shape maintenance, characterized by spheroid morphology, reduced *D*_max_/*D*_min_ ratios and diminished alpha‐granule pools. Flow cytometry and single‐platelet total internal reflection fluorescence imaging demonstrated largely preserved calcium signalling but impaired activation‐dependent shape change, dense‐granule release and integrin activation. Proteomics showed reduced alpha‐granule and lysosomal proteins alongside imbalanced regulators of actin remodelling and β‐tubulin. Phalloidin staining confirmed impaired actin cytoskeletal remodelling with reduced lamellipodia formation, while immunofluorescence revealed abnormal β1‐tubulin localization with disrupted marginal bands and reduced lysosome‐associated membrane protein 1 (LAMP1) expression. Additionally, granulocyte recruitment and migration within thrombi were impaired, suggesting broader thromboinflammatory defects. These findings suggest that combined disruption of cytoskeletal integrity and granule biogenesis underlies impaired thrombus formation in ETV6‐RT, providing mechanistic insight into the haemostatic defects associated with this disorder.

## INTRODUCTION

ETV6‐related thrombocytopenia (ETV6‐RT) is an autosomal dominant disorder caused by germline ETV6 variants.^1^, ^2^, ^3^, ^4^ Since its first description in 2015, more than 100 affected individuals from about 30 families have been reported worldwide.^5^, ^6^

Although the clinical picture is usually limited to thrombocytopenia and variable bleeding, ETV6‐RT confers a 20%–25% lifetime risk of haematological malignancies,^7^, ^8^, ^9^, ^10^ and germline ETV6 variants are found in approximately 0.8% of paediatric B‐cell acute lymphoblastic leukaemia (B‐ALL) cases.^1^, ^11^ Solid tumours have also been described in affected families,^2^, ^7^ although a causal link to ETV6 is uncertain.

ETV6 encodes a transcriptional repressor essential for haematopoiesis, containing an N‐terminal pointed (PNT) oligomerisation domain, a central regulatory domain, and an E26 transformation‐specific (ETS) deoxyribonucleic acid (DNA)‐binding domain.^11^ It regulates genes involved in transcription, cell cycle control, apoptosis, adhesion,^12^ and innate immunity,^11^ and functions in repressor complexes with SIN3 transcription regulator family member A (SIN3A), nuclear receptor co‐repressor (N‐CoR), and histone deacetylase 3 (HDAC3).^13^, ^14^ Germline variants, often in the ETS domain, impair DNA binding and nuclear localization and act in a dominant negative manner.^3^, ^4^, ^11^, ^15^ Experimental models indicate defective megakaryocyte maturation and thrombopoiesis.^7^, ^9^, ^16^

Patients typically show mild‐to‐moderate thrombocytopenia with normal mean platelet volume,^2^, ^6^, ^9^ preserved aggregation to strong agonists,^5^, ^7^, ^17^ but impaired dense‐granule secretion^6^, ^7^ and abnormal megakaryocyte morphology.^4^, ^7^ Transcriptomic data implicate dysregulated ribosome biogenesis and cytoskeletal pathways.^16^ Together, these observations suggest qualitative platelet defects driven by cytoskeletal disruption, but underlying mechanisms remain incompletely defined.

Here, we examined 12 patients with ETV6‐RT from six families and identified impaired cytoskeletal integrity and defective granule biogenesis as central mechanisms of platelet dysfunction in this disease.

## METHODS

### Clinical evaluation

This single‐centre observational study included patients with genetically confirmed ETV6‐RT, their parents and healthy donors referred to the Dmitry Rogachev National Medical Research Center. Clinical evaluation comprised detailed histories, physical examination and standardized bleeding assessment using the International Society on Thrombosis and Haemostasis Bleeding Assessment Tool (ISTH‐BAT),^18^ Pediatric Bleeding Questionnaire (PBQ),^19^ and World Health Organization Bleeding Scale (WHO BS).^20^

### Ex vivo thrombus formation

Thrombus formation was analysed in parallel‐plate flow chambers coated with fibrillar collagen at a shear rate of 100 s^−1^, as described.^21^ Thrombi were visualized using a Nikon Eclipse Ti‐E inverted microscope.

### Flow cytometry

Flow cytometry was performed as described.^22^, ^23^ Citrated whole blood (3.8% v/v) was diluted 1:20 in Tyrode's buffer, and platelets were stimulated with 10 μg/mL collagen‐related peptide (CRP) plus 12.5 μM proteinase‐activated receptor 1 (PAR1)‐activating peptide (PAR1‐AP) SFLLRN for 10 min. Samples were stained with anti‐CD42b, anti‐CD61, PAC‐1 (monoclonal antibody specifically recognising activated glycoprotein IIb/IIIa), anti‐CD62p and Annexin V; dense‐granule content and release were assessed using 10 μM mepacrine. Data were acquired on a NovoCyte flow cytometer.

For platelet calcium mobilization, platelets were loaded with 2 μM Fura‐Red AM and stimulated with adenosine diphosphate (ADP; 2 μM), SFLLRN (5 μM) or fucoidan (100 μg/mL). Fluorescence was measured on a FACSCanto II flow cytometer, and cytosolic Ca^2+^ concentrations were calculated using the Grynkiewicz equation.^24^

### Transmission electron microscopy

Transmission electron microscopy (TEM) was performed using glutaraldehyde/osmium fixation, resin embedding, ultramicrotomy and uranyl/lead staining, followed by quantitative morphometric analysis as previously described,^25^ with 20 healthy donors serving as controls.

### Proteomics studies

Platelets were isolated by centrifugation^26^ and stored in liquid nitrogen. Tryptic peptides were analysed by liquid chromatography‐tandem mass spectrometry (LC‐MS/MS) on a Q‐Exactive HF‐X system; proteins were identified in MaxQuant with label‐free quantification (Figure S1).^26^ Differential expression and protein–protein interaction (PPI) analyses were performed using standard pipelines, including the Search Tool for Retrieval of Interacting Genes/Proteins (STRING),^27^ and statistical testing with the Mann–Whitney *U*‐test followed by false discovery rate (FDR) correction.

### Platelet spreading and F‐actin staining

Blood was perfused over fibrinogen‐coated flow chambers^28^ to generate a monolayer of adherent platelets, which were further washed, fixed, permeabilized and stained with Phalloidin‐647 and Hoechst 33342. Spreading morphology was quantified by lamellipodia presence and circularity (4*πS*/*P* ^2^).^29^, ^30^

### Immunofluorescence staining of peripheral blood films

Immunofluorescence microscopy on standard peripheral blood films was performed as previously described.^31^, ^32^

### Granulocyte recruitment and migration within thrombi

Hirudin‐anticoagulated blood was perfused through collagen‐coated flow chambers at 100 s^−1^. Thrombus formation and granulocyte migration were recorded in differential interference contrast (DIC)/epifluorescence mode on a Nikon Eclipse Ti‐E microscope. Automated particle tracking in ImageJ using a Python‐based algorithm,^33^ followed by manual verification, was used to quantify granulocyte trajectories and velocities.^21^

### Statistical analysis

Statistical analysis was performed using Python 3.8 and GraphPad Prism 8. Data were compared using the two‐tailed Mann–Whitney *U*‐test, with *p* < 0.05 considered statistically significant.

Detailed descriptions of experimental procedures, including additional assays, are provided in the Supplementary Methods.

## RESULTS

### Patient characteristics

We identified 12 patients (seven paediatric, five adult) from six families with ETV6‐RT carrying six distinct ETV6 variants (Table 1). Four variants (c.1148A>G, p.H383R; c.1105C>T, p.R369W; c.641C>T, p.P214L; gross deletion in exon 5) were previously reported,^4^, ^7^, ^9^, ^17^ whereas two (c.1172A>G, p.Y391C; c.1192C>G, p.L398V) were absent from public databases and were predicted deleterious by in silico tools (Table S1). Both novel variants co‐segregated with thrombocytopenia. Variants in families 1–4 mapped to the ETS domain, while those in families 5 and 6 affected the central regulatory domain. Median age was 10 years (5–18) for the paediatric patients and 43 years (36–48) for the adults.

**TABLE 1:** Patient characteristics.

Family	ETV6 variant^a^	Exon^b^	Domain	ACMG classification^c^	Individual	Sex	Age,^d^ years	Platelet count^e^ (range), ×10^9^/L	MPV,^f^ fL	ANC,^g^ ×10^9^/L	ISTH BAT^h^	PBQ^i^	WHO BS^j^	Bleeding phenotype	Haematological malignancies	Infections	Other medical conditions
1	c.1148A>G, p.H383R	6	ETS	LP	1.1	F	45	140–279^k^	NA	2.3	0	0	0	—	—	—	—
					1.2	M	10	90–100	9.3	2.9	1	1	1	Post‐traumatic petechiae (from minimal trauma), ecchymoses on limbs, nasal bleeds (mostly during infections), gingival bleeding	Common B‐ALL (B‐II) at age of 2 years	Frequent upper respiratory tract infections (six or more episodes per year), otitis media	—
2	c.1105C>T, p.R369W	6	ETS	LP	2.1	F	36	87–160	NA	3.1	0	0	0	—	—	—	—
					2.2	F	10	60–140	7.8	3.2	1	1	1	Petechiae, bleeding during teething, ecchymoses, post‐injection bleeding and ecchymoses	—	—	Type 1 diabetes mellitus
3	c.1172A>G, p.Y391C^l^	7	ETS	LP	3	F	5	55–127	8.6	2.4	3	3	1	Petechiae, bleeding in sclera after swimming	—	—	Asymptomatic microhaematuria
4	c.1192C>G, p.L398V^m^	7	ETS	LP	4.1	F	48	96–136	NA	2.8	1	1	1	Predisposition for haematomas, gingival bleedings	—	—	—
					4.2	M	18	11–125	11.6	2.4	9	9	2	Petechiae from birth, ecchymoses, post‐traumatic haematomas on legs, gingival bleedings, episodes of blood in stool	—	—	—
5	c.641C>T, p.P214L	5	CRD	LP	5.1	F	43	90–156	NA	5.6	0	−2	0	—	—	—	Chronic obstructive pulmonary disease, exogenous obesity
					5.2	M	7	30–123	NA	4.1	4	4	1	Nasal bleedings, spontaneous ecchymoses on lower limbs	—	Frequent upper respiratory tract infections, cervical lymphadenitis, cervical lymph node abscess	PTEN hamartoma tumour syndrome (het. *PTEN* c.545dup p.Leu182PhefsTer8)
6	Gross deletion of exon 5	5	CRD	P	6.1	M	43	55–100	NA	2.7	0	0	0	—	—	—	—
					6.2	M	9	18–140	NA	1.9	5	5	3	Post‐traumatic ecchymoses (from minimal trauma), few petechiae, one episode of severe nasal bleeding (during influenza B infection)	—	—	—
					6.3	M	12	55–111	9.7	3.2	0	0	0	—	—	—	—

Abbreviations: ANC, absolute neutrophil count; B‐ALL, B‐cell acute lymphoblastic leukaemia; CRD, central regulatory domain; ETS, erythroblast transformation specific; F, female; ISTH BAT, International Society on Thrombosis and Haemostasis Bleeding Assessment Tool; M, male; MPV, mean platelet volume; NA, not available; PBQ, Pediatric Bleeding Questionnaire; WHO BS, World Health Organization Bleeding Scale.

^a^ Nucleotide A of the start codon in the ETV6 cDNA (GenBank accession NM_001987.5) is indicated as nucleotide +1.

^b^ According to the RefSeq transcript NM_001987.5.

^c^ Classification according to American College of Medical Genetics and Genomics (ACMG)^34^: LP, likely pathogenic; P, pathogenic.

^d^ Age at enrolment.

^e^ Reference range: 150–450 × 10^9^/L.

^f^ Mean platelet volume; institutional reference range: 9.0–13.0 fL.

^g^ Absolute neutrophil count; institutional reference range: 1.5–7.8 × 10^9^/L.

^h^ International Society on Thrombosis and Hemostasis—Bleeding Assessment Tool^18^; abnormal if ≥3 in children, ≥4 in adult males, ≥6 in adult females.^35^

^i^ Pediatric Bleeding Questionnaire^19^; abnormal if ≥3 in children.^35^, ^36^

^j^ World Health Organization Bleeding Scale^20^; grade 0, no bleeding; grade 1, cutaneous bleeding only; grade 2, mild blood loss; grade 3, gross blood loss; and grade 4, debilitating blood loss.^20^

^k^ Adult values; childhood data not available.

^l,m^ Novel variants.

Most patients had mild‐to‐moderate thrombocytopenia. Patient 1.1 had predominantly normal platelet counts with only intermittent mild thrombocytopenia documented in adulthood. The bleeding phenotype was moderate in children and mild in adults: median ISTH‐BAT, PBQ and WHO BS scores were 3 (1–9), 3 (0–9) and 1 (0–3) in paediatric patients and 0 (0–1), 0 (−2 to 1) and 0 (0–1) in adults respectively (abnormal ISTH‐BAT ≥3 in children, ≥4 in adult males, ≥6 in adult females^35^; abnormal PBQ ≥3^35^, ^36^; abnormal WHO BS ≥1^20^). The most common paediatric bleeding symptoms were petechiae, ecchymoses and epistaxis, with gingival bleeding and post‐traumatic haematomas also frequently reported.

Patient 1.2 developed B‐ALL at age 2 years, achieved complete remission after standard chemotherapy and remains in sustained remission for over 7 years; haematopoietic stem cell transplantation was not performed; and the germline ETV6 variant was identified in remission. None of the other participants has developed haematological malignancy to date.

Two patients (Patients 1.2 and 5.2) showed features of immunodeficiency. Patient 1.2 had frequent bacterial respiratory infections (>6 per year) and recurrent otitis media, with normal white blood (WBC) and absolute neutrophil (ANC) counts but decreased immunoglobulin A (IgA; <0.15 g/L; reference 0.90–1.90 g/L) and immunoglobulin G (IgG; 1.74 g/L; reference 8.70–11.70 g/L). Patient 5.2 had recurrent upper respiratory tract infections, cervical lymphadenitis, lymph node abscesses and a neck fistula; WBC, ANC and IgA were normal, with mildly reduced IgG (6.79 g/L).

Patient 5.2 was diagnosed with phosphatase and tensin homolog (PTEN) hamartoma tumour syndrome with a heterozygous *PTEN* c.545dup, p.L182fs variant. Additional comorbidities included type 1 diabetes mellitus (Patient 2.2), microhaematuria (Patient 3) and exogenous obesity (Patient 5.1).

### Selective platelet activation defects and reduced thrombus formation

Although platelet counts were only mildly to moderately reduced in most patients, 7 of 12 experienced bleeding episodes of varying severity, irrespective of platelet count (Table 1). Given that, in the absence of platelet dysfunction, only severe thrombocytopenia typically causes bleeding,^37^ we performed a comprehensive assessment of platelet function.

**FIGURE 1:**
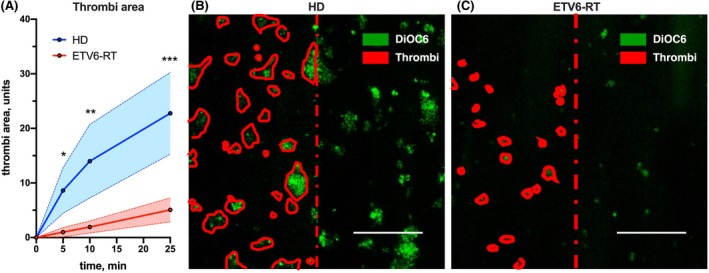
Collagen‐induced platelet thrombus formation in ETV6‐related thrombocytopenia (ETV6‐RT). (A) Platelet thrombus area measured 5, 10 and 25 min after the start of perfusion in six paediatric patients with ETV6‐RT and 10 paediatric healthy donors (HDs). Blue curves represent HDs and red curves represent ETV6‐RT; solid lines indicate the mean thrombus area and dashed lines indicate the standard deviation (SD). Statistical comparisons between groups at each time point were performed using the Mann–Whitney *U*‐test. *p < 0.05; **p < 0.01; ***p < 0.001; absence of a symbol indicates no statistically significant difference. (B, C) Representative microscopy images of collagen‐induced thrombus growth in a HD (B) and an ETV6‐RT patient (C). Scale bars represent 50 μm. Platelets were labelled with 3,3'‐dihexyloxacarbocyanine iodide (DiOC‐6; green). Red outlines in the left half of each image indicate segmented thrombi.

Light transmission aggregometry showed largely preserved aggregation to collagen, PAR1‐AP and ristocetin (Figure S2). Aggregation to ADP and adrenaline was mildly reduced in Patients 1.2 and 4.2 but normal in the others. In contrast, collagen‐induced thrombus formation was consistently reduced in both paediatric (Figure 1; Figure S3) and adult (Figures S3 and S4) patients, a defect not explained by the mild‐to‐moderate thrombocytopenia present in most individuals.^21^, ^23^

To distinguish a global clotting defect from a platelet‐specific abnormality, we evaluated clot viscoelasticity by thromboelastography. Maximum clot amplitude was consistently reduced, suggesting impaired platelet contribution to clot strength^38^, ^39^ (Figure S5).

**FIGURE 2:**
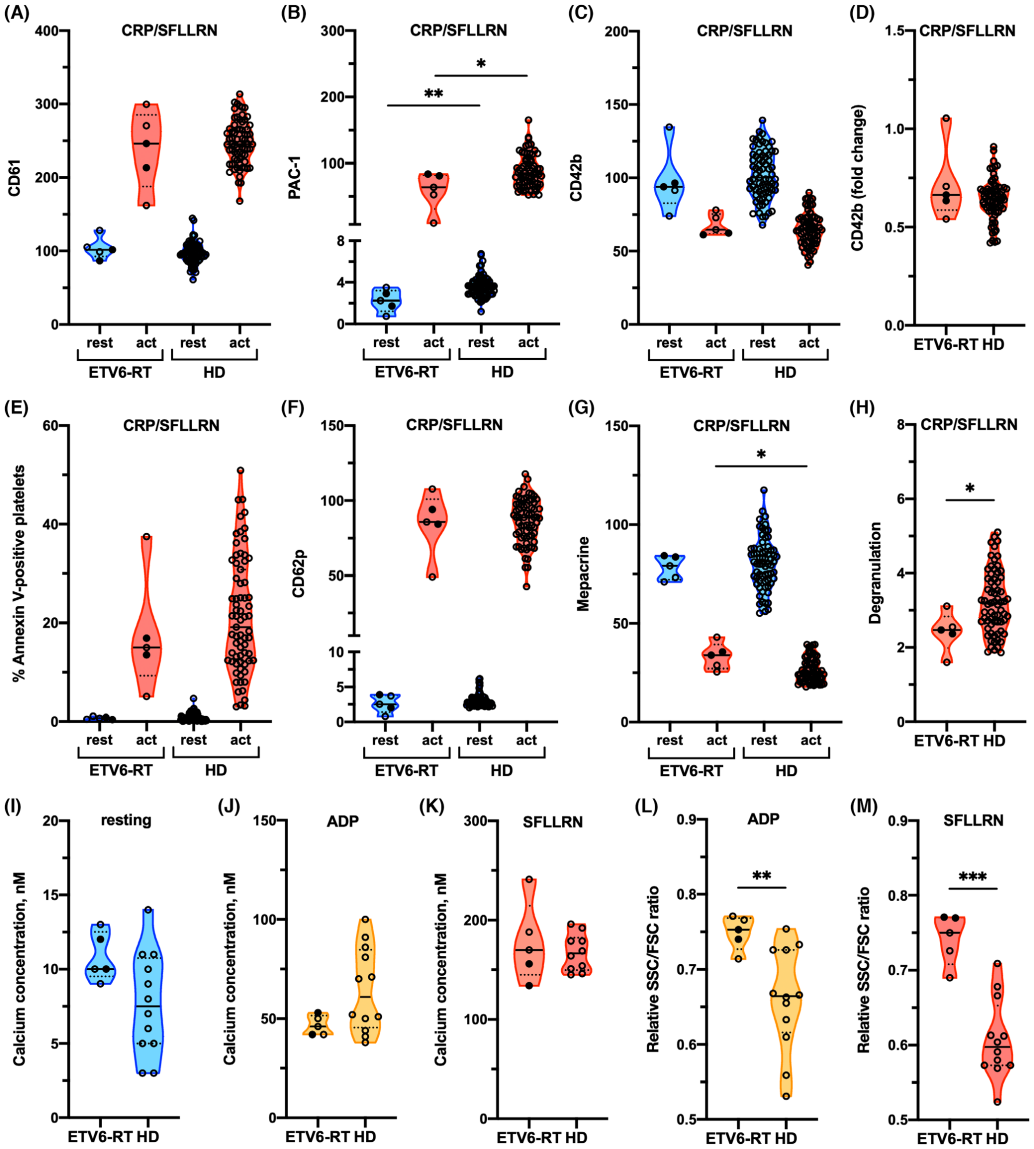
Flow cytometry analysis of platelet function in paediatric patients with ETV6‐related thrombocytopenia (ETV6‐RT). Platelet functional parameters were assessed in paediatric ETV6‐RT patients and healthy donors (HDs) under resting conditions (A–H, ‘rest’; I), after strong dual‐agonist stimulation with 10 μg/mL collagen‐related peptide (CRP) plus 12.5 μM PAR1‐activating peptide (SFLLRN) (A–H, ‘act’) and after stimulation with 2 μM ADP (J, L) or 5 μM SFLLRN (K, M). (A) Surface expression of glycoprotein IIIa (GPIIIa) (CD61) in resting platelets and after dual‐agonist stimulation. (B) Expression of active GPIIb/IIIa (PAC‐1 binding) in resting platelets and after dual‐agonist stimulation. (C) Surface expression of GPIb (CD42b) in resting platelets and after dual‐agonist stimulation. (D) Activation‐induced CD42b fold change (ratio of CD42b MFI after dual‐agonist stimulation to resting MFI), reflecting GPIb loss from the platelet surface. (E) Percentage of Annexin V‐positive (procoagulant) platelets in resting conditions and after dual‐agonist stimulation. (F) P‐selectin (CD62p) expression in resting platelets and after dual‐agonist stimulation. (G) Dense‐granule content in resting platelets and after dual‐agonist stimulation, measured as mepacrine fluorescence intensity. (H) Dense‐granule release after dual‐agonist stimulation, expressed as the activation‐induced change in mepacrine signal (degranulation index; ratio of resting to post‐stimulation fluorescence). (I) Cytosolic calcium concentration in resting platelets. (J, K) Calcium mobilization following stimulation with ADP (J) or SFLLRN (K). (L, M) Platelet shape change after stimulation with ADP (L) or SFLLRN (M), expressed as the relative change in side scatter (SSC)/forward scatter (FSC) ratio. Data are presented as violin plots showing the distribution of values, with width representing data density. Individual measurements are shown as circles, data points from individuals carrying novel ETV6 variants (Patients 3 and 4.2) are highlighted as black circles; solid horizontal lines indicate the median; and dotted horizontal lines indicate the 25th and 75th percentiles. Statistical comparisons were performed using the Mann–Whitney *U*‐test. *p < 0.05; **p < 0.01; ***p < 0.001; absence of a symbol indicates no statistically significant difference.

To further delineate the underlying platelet defects, we analysed platelet functional responses by flow cytometry. Baseline glycoprotein IIIa (GPIIIa, CD61) and GPIb (CD42b) expression was comparable to controls in children (Figure 2A,C) but elevated in adults (Figure S6A,C), whereas the activated fraction of GPIIb/IIIa, assessed by PAC‐1 binding, was markedly reduced in both age groups (Figure 2B; Figure S6B). P‐selectin (CD62p) exposure in resting platelets (Figure 2F; Figure S6F), dense‐granule content by mepacrine (Figure 2G; Figure S6G), Annexin V‐positive platelets at rest (Figure 2E; Figure S6E) and basal cytosolic calcium (Figure 2I; Figure S6I) were all within control ranges, arguing against increased platelet death as the primary cause of thrombocytopenia.^11^

After strong stimulation with 10 μg/mL CRP and 12.5 μM SFLLRN, GPIIb/IIIa activation remained consistently reduced in both paediatric (Figure 2B) and adult (Figure S6B) patients. Activation‐induced GPIb shedding (CD42b mean fluorescence intensity (MFI) fold change) was preserved in children (Figure 2D) and increased in adults (Figure S6D). Procoagulant platelet formation was normal in paediatric patients (Figure 2E) and increased in adults (Figure S6E). Dense‐granule release upon activation was markedly reduced in both age groups (Figure 2H; Figure S6H).

With weak stimulation by 2 μM ADP, cytosolic calcium mobilization was preserved in paediatric patients (Figure 2J) but reduced in a subset of adults (Figure S6J). No differences were observed after stronger stimulation with 5 μM SFLLRN (Figure 2K; Figure S6K). Consistently, single‐platelet total internal reflection fluorescence imaging^28^ showed broadly similar calcium dynamics between ETV6‐RT patients and controls (Figure S7).

### Altered cytoskeletal integrity and defective granule biogenesis

**FIGURE 3:**
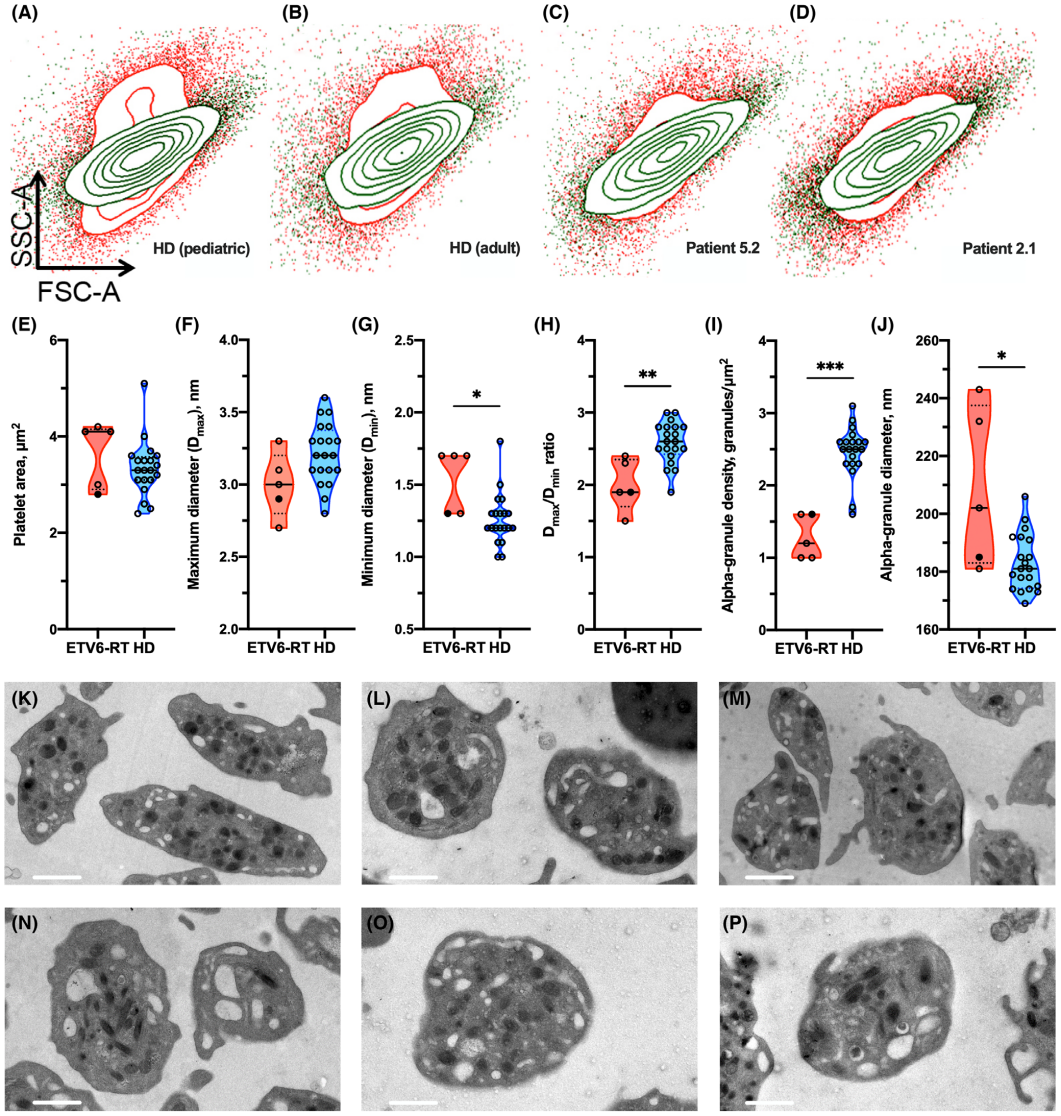
Light‐scattering and ultrastructural analysis of platelets in ETV6‐related thrombocytopenia (ETV6‐RT). (A–D) Flow cytometry side scatter (SSC) versus forward scatter (FSC) of platelets in the resting state (red) and following activation with 5 μM SFLLRN (green) for paediatric (A) and adult (B) healthy donors (HDs) and paediatric (C) and adult (D) patients with ETV6‐RT. (E–J) Quantitative morphometric analysis of platelets by transmission electron microscopy. Morphometric parameters were assessed in platelets from five ETV6‐RT patients (Patients 1.2, 3, 5, 6.2 and 6.3) and 20 HDs, as means of 29–100 platelets evaluated per individual: Platelet area (E); maximum platelet diameter (F); minimum platelet diameter (G); maximum to minimum platelet diameter ratio (H); alpha‐granule density (number of alpha‐granules per platelet area) (I); alpha‐granule diameter (J). Data are presented as violin plots showing the distribution of values, with width representing data density. Individual measurements are shown as circles, with ETV6‐RT patients in red and HD in blue, and data points from the individual carrying a novel ETV6 variant (Patient 3) are highlighted as black circles; solid horizontal lines indicate the median, and dotted horizontal lines indicate the 25th and 75th percentiles. Statistical comparisons were performed using the Mann–Whitney *U*‐test. *p < 0.05; **p < 0.01; ***p < 0.001; absence of a symbol indicates no statistically significant difference. (K–P) Representative transmission electron micrographs of platelets from a HD (K) and five individuals with ETV6‐RT: Patient 1.2 (L), Patient 3 (M), Patient 5 (N), Patient 6.2 (O) and Patient 6.3 (P). Scale bars represent 1 μm.

Platelet shape change, assessed by the side scatter (SSC)/forward scatter (FSC) ratio,^40^, ^41^ was significantly reduced after both weak (Figure 2L; Figure S6L) and strong (Figure 2M; Figure S6M) stimulation. Together with abnormal activation‐induced changes in light‐scattering profiles (Figure 3A–D), these findings indicate impaired morphological remodelling during platelet activation, suggesting disturbed cytoskeletal dynamics in ETV6‐RT.^42^

TEM morphometry demonstrated that ETV6‐RT platelets exhibited a more rounded, spheroid‐like morphology, with a significantly reduced maximum to minimum diameter ratio (*D*_max_/*D*_min_) (Figure 3E–H,K–P). Alpha‐granule analysis revealed fewer granules per platelet (Figure 3I) and a higher proportion of morphologically abnormal granules, including elongated, enlarged and small granule‐like organelles (Figure 3J–P). All patients displayed an expanded open canalicular system. These findings are consistent with altered membrane remodelling and platelet shape maintenance in ETV6‐RT.

**FIGURE 4:**
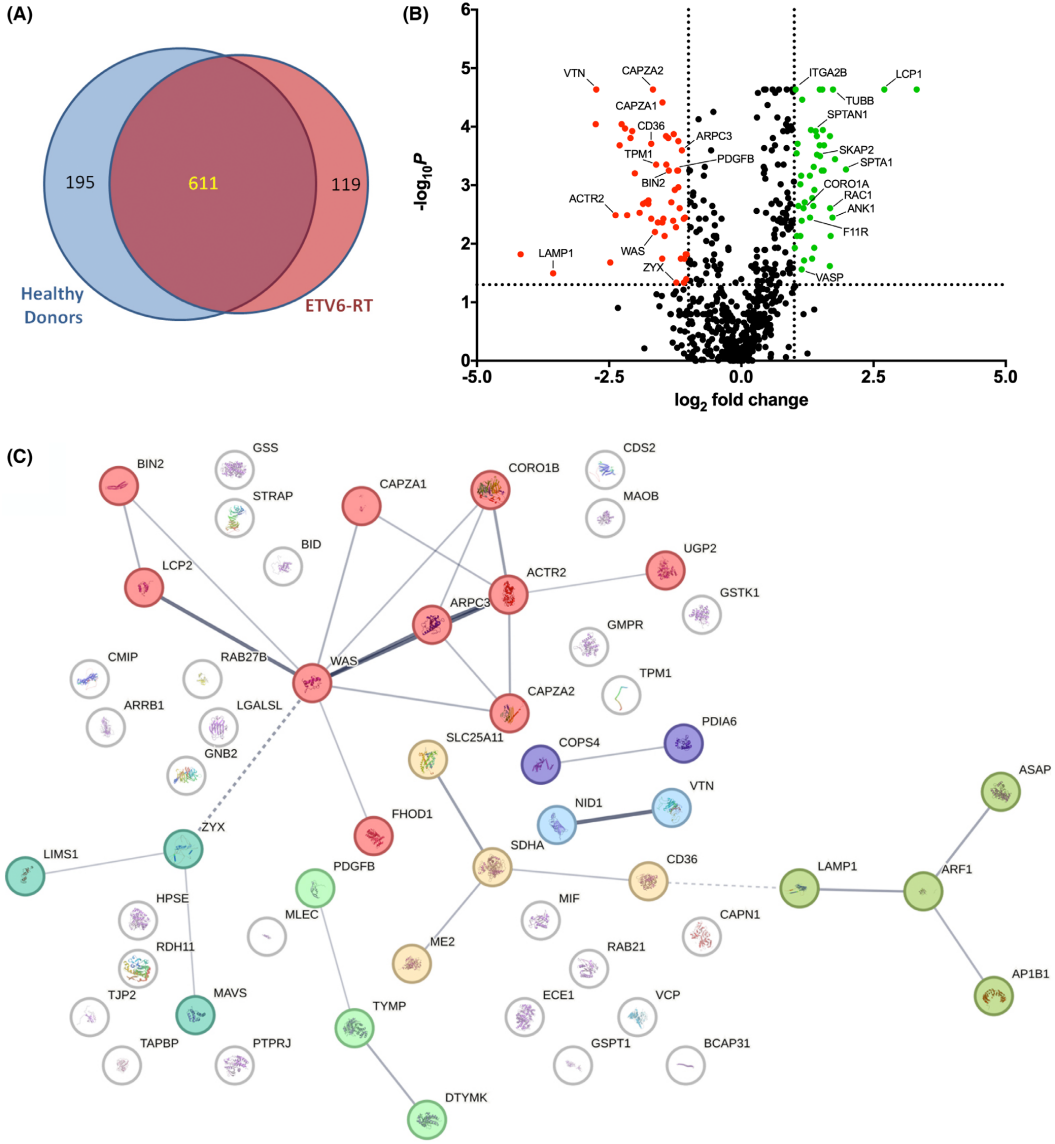
Effects of germline ETV6 mutations on platelet proteomic profile. (A) Venn diagram of proteins identified in platelets from healthy donors and ETV6‐related thrombocytopenia (ETV6‐RT) patients. (B) Volcano plot showing differential protein expression between ETV6‐RT and healthy controls (restricted to 611 proteins detected in both groups). The plot depicts log_2_ fold changes versus −log_10_ *p*‐values (Mann–Whitney *U*‐test with FDR correction). Red dots indicate proteins significantly downregulated in ETV6‐RT (log_2_ fold change ≤−1.0; *p* < 0.05); green dots indicate proteins significantly upregulated (log_2_ fold change ≥1.0; *p* < 0.05); black dots represent non‐significant proteins. (C) STRING database (string‐db.org) network analysis of differentially expressed proteins. Edge thickness reflects confidence of protein–protein associations, and node colours indicate Markov cluster algorithm (MCL) clusters.

Given ETV6 regulation of cytoskeletal and signalling networks,^12^ we compared platelet proteomes between six ETV6‐RT patients (1.1, 1.2, 2.1, 2.2, 4.1, 4.2) and six age‐matched healthy donors (Table S2). In total, 806 proteins were identified in controls and 730 in patients, with 611 detected in all samples (Figure 4A). Ninety‐nine differentially expressed proteins (DEPs) were identified (54 downregulated, 45 upregulated; Figure 4B; Table S3), with no significant difference between paediatric and adult patients (not shown).

Network analysis of DEPs revealed significantly more PPIs than expected (237 observed vs. 102 expected; PPI enrichment *p* < 10^−16^), with strong enrichment for pathways regulating actin cytoskeleton organization and cell–substrate adhesion (FDR <10^−5^). Downregulated proteins included actin‐related protein 2/3 (Arp2/3) complex subunits (actin‐related protein 2 [ACTR2], actin‐related protein 2/3 complex subunit 3 [ARPC3]) and Wiskott‐Aldrich syndrome protein (WAS), whereas upregulated proteins comprised Ras‐related C3 botulinum toxin substrate 1 (RAC1), proto‐oncogene tyrosine‐protein kinase Src (SRC), vasodilator‐stimulated phosphoprotein (VASP) and regulators of actin–myosin interactions (caldesmon [CALD1], Cas scaffolding protein family member 4 [CASS4]) (Figure 4B,C). Additional alterations were observed in proteins involved in actin dynamics and motility (downregulated coronin‐1B [CORO1B], formin homology 2 domain‐containing protein 1 [FHOD1]; upregulated coronin‐1A (CORO1A)) and adhesion signalling (downregulated platelet glycoprotein 4 [CD36], LIM and senescent cell antigen‐like‐containing domain protein 1 [LIMS1], nidogen‐1 [NID1], vitronectin [VTN]; upregulated Rho GDP‐dissociation inhibitor 1 [ARHGDIA], CASS4, RAC1). The β‐tubulin chain (TUBB) was also significantly upregulated, indicating microtubule cytoskeleton alterations.

STRING‐based^27^ enrichment analysis of the 611 proteins detected in all samples showed significant functional associations, including enrichment with large‐scale blood cell genetics datasets^43^ (enrichment score 8.85, FDR = 0.0092) and with pathways linked to sphingolipid regulation and proplatelet formation^44^ (enrichment score 7.45, FDR = 0.0092). These data further implicate cytoskeletal and metabolic pathways in the pathogenesis of ETV6‐RT.

**FIGURE 5:**
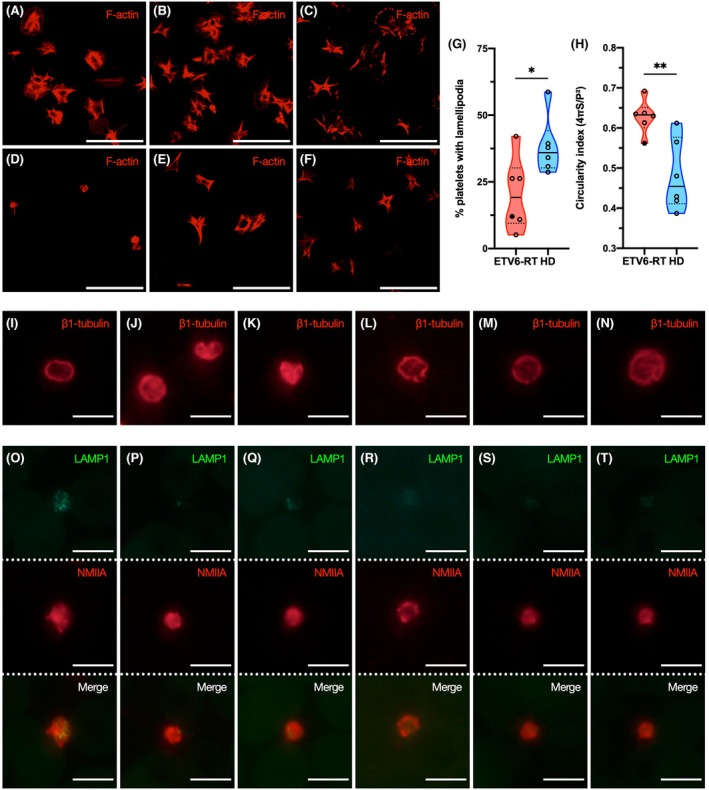
Platelet spreading, cytoskeletal and lysosomal protein organization in ETV6‐related thrombocytopenia (ETV6‐RT). (A–F) Representative fluorescence microscopy images of spread platelets stained for F‐actin (Phalloidin‐647) from a healthy donor (HD) (A) and ETV6‐RT patients: Patient 1.1 (B), Patient 1.2 (C), Patient 4.2 (D), Patient 6.1 (E), Patient 6.3 (F). Scale bars represent 20 μm. (G, H) Quantitative morphometric analysis of platelets from six ETV6‐RT patients (Patients 1.1, 1.2, 4.2, 6.1, 6.2, 6.3) and six HDs, as means of 91–276 platelets evaluated per individual: Percentage of platelets exhibiting lamellipodia (G); circularity index (H) calculated as 4*πS*/*P* ^2^, where *S* = platelet area and *P* = perimeter. Data are presented as violin plots showing the distribution of values, with width representing data density. Individual measurements are shown as circles (ETV6‐RT patients in red, HDs in blue); data points from the patient carrying a novel ETV6 variant (Patient 4.2) are highlighted as black circles. Solid horizontal lines indicate the median; dotted horizontal lines indicate the 25th and 75th percentiles. Statistical comparisons were performed using the Mann–Whitney *U*‐test. *p < 0.05; **p < 0.01. (I–N) Representative immunofluorescence images of peripheral blood films stained for β1‐tubulin from HD (I) and ETV6‐RT patients: Patient 1.1 (J), Patient 1.2 (K), Patient 4.2 (L), Patient 6.1 (M), Patient 6.3 (N). Scale bars, 5 μm. (O–T) Representative dual immunofluorescence images of peripheral blood films stained for LAMP1 (green) and non‐muscle myosin IIA (NMIIA; red) from HD (O) and ETV6‐RT patients: Patient 1.1 (P), Patient 1.2 (Q), Patient 4.2 (R), Patient 6.1 (S), Patient 6.3 (T). Scale bars, 5 μm.

Analysis of F‐actin organization in adherent platelets (Figure 5A–F) revealed markedly impaired spreading morphology, with significantly reduced lamellipodia formation (Figure 5G) and increased circularity (Figure 5H). Although overall filopodia formation did not differ significantly between patients and controls (Figure S8), some patients (notably 4.2 and 6.2) showed markedly reduced filopodia counts, with >33% of platelets lacking filopodia. These defects corroborate proteomic evidence of dysregulated actin remodelling and support cytoskeletal disruption as a central mechanism of platelet dysfunction in ETV6‐RT.

Immunofluorescence microscopy provided complementary evidence of abnormal tubulin cytoskeleton organization (Figure 5I–N). In healthy platelets, β1‐tubulin formed a well‐organized peripheral ring (Figure 5I), whereas in most ETV6‐RT patients, notably 1.1, 1.2 and 6.3, β1‐tubulin displayed a markedly disorganized distribution (Figure 5J–N), suggesting underlying microtubule cytoskeleton abnormalities that may additionally contribute to altered platelet shape maintenance.

Proteomics also revealed significant downregulation of alpha‐granule and lysosomal proteins (e.g. platelet‐derived growth factor subunit B [PDGFB], lysosome‐associated membrane protein 1 [LAMP1]) together with regulators of protein trafficking and granule biogenesis (Figure 4B,C). Immunofluorescence confirmed markedly reduced LAMP1 expression in all patients (Figure 5O–T; Figure S9). Along with the reduced alpha‐granule content observed by TEM (Figure 3I,K–P), these findings indicate defective lysosomal/alpha‐granule biogenesis and trafficking in ETV6‐RT.

### Impaired granulocyte recruitment and migration within thrombi

**FIGURE 6:**
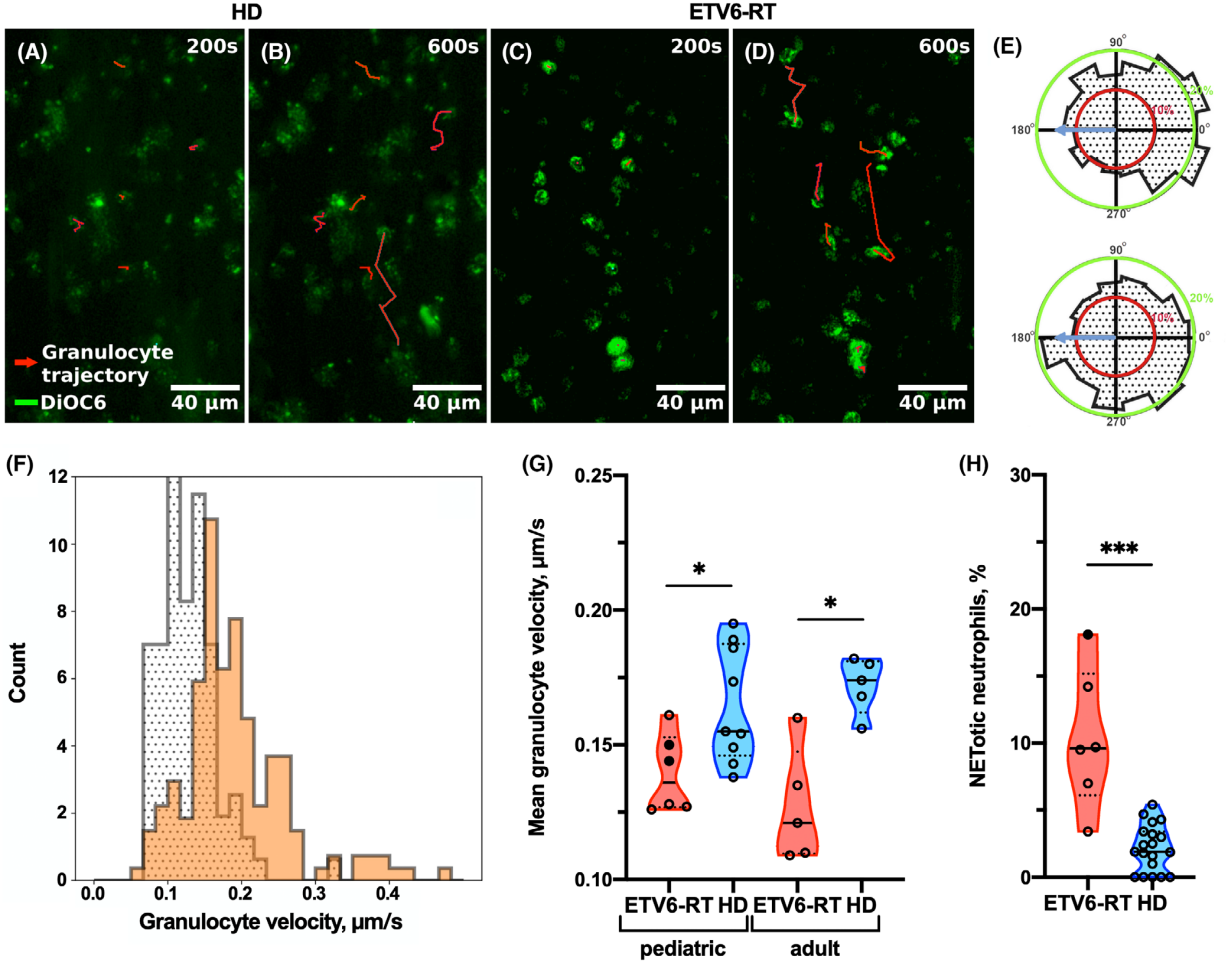
Granulocyte recruitment into thrombi and NETosis in patients with ETV6‐related thrombocytopenia (ETV6‐RT). (A–D) Representative microscopy images of granulocyte recruitment and migration within developing thrombi for healthy donors (HDs) (A, 200 s; B, 600 s) and ETV6‐RT patients (C, 200 s; D, 600 s). Granulocyte trajectories are shown in red; platelets are labelled with DiOC‐6 (green). (E) Polar plots of granulocyte movement directions in ETV6‐RT patients. The blue arrow indicates flow direction. Granulocytes predominantly migrated against the flow in some patients (upper panel, Patient 4.2) or independently of flow in others (lower panel, Patient 2.1). (F) Distribution of velocities of thrombus‐associated granulocytes in an ETV6‐RT patient (dots; Patient 2.2) compared with a representative HD (orange). (G) Mean velocities of thrombus‐associated granulocytes in adult and paediatric ETV6‐RT patients and healthy individuals. (H) Percentage of NETotic neutrophils in leucocyte‐rich plasma samples from patients with ETV6‐RT and healthy controls; neutrophils were identified by double‐positive immunostaining for myeloperoxidase and human neutrophil elastase. (G, H) Data are presented as violin plots showing the distribution of values, with width representing data density. Individual measurements are shown as circles, with ETV6‐RT patients in red and HD in blue; data points from individuals carrying novel ETV6 variants (Patients 3 and 4.2 in G; Patient 4.2 in H) are highlighted as black circles. Solid horizontal lines indicate the median, and dotted horizontal lines indicate the 25th and 75th percentiles. Statistical comparisons were performed using the Mann–Whitney *U*‐test. *p < 0.05; ***p < 0.001; absence of a symbol indicates no statistically significant difference. NET, neutrophil extracellular traps.

Germline ETV6 mutations affect multiple haematopoietic lineages^2^, ^16^ and, beyond thrombocytopenia, have been associated with neutropenia, recurrent infections,^4^, ^7^, ^17^ and dysregulated inflammatory signalling and leucocyte migration,^11^ suggesting an impact on innate immune function. Because granulocytes play a central role in early thromboinflammation through their recruitment within developing thrombi, we examined granulocyte migration trajectories in an ex vivo microfluidic model^21^ (Figure 6A–D).

We first analysed the directional component of migration by measuring, for each tracked cell and frame, the angle (*θ*) between movement direction and flow. In healthy donors, granulocyte migration was consistently biased against the direction of flow, whereas trajectories from ETV6‐RT patients (1.1, 2.1, 2.2) showed no stable directional preference (Figure 6E; Figure S10A–F), indicating disrupted thrombus‐directed chemotaxis. We then compared the spatial distribution of granulocytes relative to thrombi with randomly generated trajectories: in 80% of healthy donors, observed tracks were significantly closer to thrombi than random simulations, whereas among the eight ETV6‐RT patients analysed, this pattern was detected only in one (Patient 5.1) (Figure S10G).

Next, we quantified the motility of thrombus‐associated granulocytes and found significantly reduced mean velocities in ETV6‐RT patients compared with healthy controls (Figure 6F,G). Among the four patients with both TEM and granulocyte migration data, those with the lowest alpha‐granule content and abnormally enlarged alpha‐granules (Patients 1.2 and 6.2) exhibited the lowest mean granulocyte velocities, whereas patients with the highest alpha‐granule load and normal alpha‐granule size (Patients 3 and 5.2) showed the highest granulocyte motility (Figure S11), consistent with the concept that loss of platelet alpha‐granule chemoattractant cargo may attenuate granulocyte chemotaxis towards activated platelets.^45^, ^46^, ^47^, ^48^

Additionally, most ETV6‐RT patients displayed increased neutrophil extracellular traps (NET) formation compared with the healthy controls (Figure 6H). Together, these findings show that germline ETV6 mutations are associated with attenuated granulocyte recruitment and migration within thrombi and heightened NETosis, pointing to a possible broader dysregulation of granulocyte responses in the thromboinflammatory setting.

## DISCUSSION

ETV6‐RT is a disorder in which bleeding often appears disproportionate to mild thrombocytopenia, suggesting a qualitative platelet defect. Previous studies have implicated impaired megakaryocyte maturation and cytoskeletal dysregulation in abnormal platelet production in ETV6‐RT.^7^, ^9^, ^16^ Here, we demonstrate that ETV6‐RT is also characterized by defective thrombus formation, selective impairment of platelet activation responses, abnormal cytoskeletal organization and granule biogenesis, together providing a mechanistic explanation for this bleeding phenotype.^37^

Our cohort comprised 12 patients carrying six ETV6 variants, including two novel (p.Y391C and p.L398V). Both variants showed phenotypic concordance with established ETV6‐RT mutations across functional assays and co‐segregated with thrombocytopenia, providing functional support for their pathogenicity. Bleeding symptoms were more pronounced in paediatric patients than in adults, consistent with previous reports.^7^, ^9^, ^17^ Collagen‐induced thrombus formation was markedly reduced in all patients, independent of platelet count and despite largely preserved aggregation responses (Figure 1; Figures S2–S4).

At the single‐platelet level, surface glycoprotein expression, dense‐granule content and calcium signalling were largely preserved (Figure 2A,C,G,I–K; Figures S6A,C,G,I–K and S7). In contrast, GPIIb/IIIa activation and dense‐granule release were consistently impaired (Figure 2B,H; Figure S6B,H), indicating defective cytoskeleton‐dependent platelet activation.

Activation‐induced platelet shape change was significantly blunted (Figure 2L,M; Figure S6L,M). Altered FSC–SSC profiles and TEM morphometry showing reduced *D*_max_/*D*_min_ ratios further supported dysregulated cytoskeletal control of platelet shape (Figure 3A–H,K–P). These findings align with prior reports of cytoskeletal abnormalities in ETV6‐RT^31^, ^49^, ^50^, ^51^ and resemble tubulinopathy‐associated platelet defects.^52^

Quantitative proteomics provided system‐level evidence for dysregulation of cytoskeletal pathways, with enrichment of proteins involved in actin filament organization and cell–substrate adhesion (Figure 4B,C; Table S3). These findings were functionally validated by impaired platelet spreading with reduced lamellipodia formation and increased circularity, demonstrating defective actin‐dependent morphological remodelling (Figure 5A–H; Figure S8). In addition, immunofluorescence revealed abnormal β1‐tubulin organization, suggesting concomitant disruption of the microtubule marginal band that may contribute to altered shape maintenance (Figure 5I–N).

Our data also indicate impaired platelet granule biogenesis. Downregulation of alpha‐granule and lysosomal proteins was supported by reduced and abnormal alpha‐granules and markedly decreased LAMP1 expression (Figures 3I–P, 4B,C and 5O–T; Figure S9), suggesting defective intracellular trafficking and granule formation that likely contribute to impaired secretion and thrombus formation.

Beyond platelets, our data suggest broader immune involvement. Two patients showed recurrent infections and reduced immunoglobulin levels despite normal ANC, consistent with previous reports in ETV6‐RT^4^, ^7^ and supporting immune dysregulation as part of the phenotype.

In an ex vivo thromboinflammation model, ETV6‐RT was associated with reduced velocities of thrombus‐associated granulocytes, loss of directional bias and diminished proximity to thrombi, indicating defective thrombus‐directed chemotaxis (Figure 6E–G; Figure S10). Two mutually not exclusive mechanisms may underlie these abnormalities. First, ETV6 has been implicated in leucocyte migration and inflammatory gene regulation,^11^ raising the possibility of cell‐intrinsic granulocyte defects affecting cytoskeletal organization, chemotactic signalling or effector functions. Consistently, most patients displayed increased NET formation, suggesting altered granulocyte effector responses (Figure 6H). Second, impaired chemotaxis towards activated platelets is a plausible contributing factor, given the reduced alpha‐granule load and the fact that alpha‐granules are a major source of neutrophil‐directed chemoattractants and growth factors (e.g. chemokine (C‐X‐C motif) ligand 7 [CXCL7], platelet factor 4, platelet‐derived growth factor) that potently promote neutrophil chemotaxis and activation.^45^, ^46^, ^47^, ^48^ The association between reduced alpha‐granule content and slower granulocyte velocities (Figure S11) supports this model, although confirmation will require larger cohorts and experiments separating granulocyte‐intrinsic from platelet‐derived effects.

In summary, ETV6‐RT is characterized by impaired thrombus formation under flow, defective platelet shape maintenance and altered cytoskeletal remodelling during activation, providing a mechanistic explanation for bleeding disproportionate to platelet count. Future studies using biomechanical approaches such as real‐time deformability cytometry may help quantify platelet biomechanical properties and clarify the translational relevance of these findings. Impaired granulocyte recruitment within thrombi further extends the phenotype to altered thromboinflammation and highlights the contribution of platelet–granulocyte crosstalk to haemostatic dysfunction.

## AUTHOR CONTRIBUTIONS

**Ivan P. Tesakov:** Conceptualization; investigation; visualization; writing – original draft; writing – review and editing; methodology. **Julia‐Jessica D. Korobkin:** Methodology; investigation; writing – review and editing. **Maria M. Golomysova:** Investigation; visualization; writing – review and editing. **Viktor G. Zgoda:** Methodology; investigation; writing – review and editing. **Daria V. Fedorova:** Conceptualization; methodology; writing – review and editing. **Ekaterina‐Iva A. Adamanskaya:** Methodology; investigation; writing – review and editing. **Anastasia A. Ignatova:** Investigation; writing – review and editing. **Igor I. Kireev:** Investigation; writing – review and editing. **Sofia V. Galkina:** Methodology; investigation; writing – review and editing. **Sergei I. Obydennyi:** Investigation; writing – review and editing. **Anna V. Pavlova:** Investigation; writing – review and editing; methodology. **Anastasia N. Sveshnikova:** Conceptualization; methodology; investigation; visualization; funding acquisition; project administration; supervision; writing – original draft; writing – review and editing. **Eugenia V. Yushkova:** Investigation; writing – review and editing. **Galina A. Novichkova:** Funding acquisition; supervision; writing – review and editing; project administration. **Pavel A. Zharkov:** Supervision; writing – review and editing. **Mikhail A. Panteleev:** Supervision; writing – review and editing.

## FUNDING INFORMATION

Platelet activity assessment was supported by the Russian Science Foundation grant 24‐15‐00387; the experiments on platelet proteomics were supported by the Russian Science Foundation grant 21‐74‐20087; the development of the ex vivo model of thromboinflammation was supported by the Russian Science Foundation grant 23‐45‐10039. Ultrastructural platelet preparation and morphometric analysis were supported by the Russian Science Foundation grant 23‐75‐10120.

## CONFLICT OF INTEREST STATEMENT

The authors declare that they have no conflicts of interest related to this work.

## ETHICS STATEMENT

The study protocol was approved by the Independent Ethics Committee of the CTP PCP RAS (approval no. 2/1‐22, dated 23 May 2022). The study was conducted in accordance with the Declaration of Helsinki and the International Conference on Harmonization Good Clinical Practice guidelines.

## PATIENT CONSENT

Written informed consent was obtained from all study participants or their legal guardians.

## Supporting information


Data S1.

Data S2.

## Data Availability

All data generated or analysed during this study are included in the main text and Supporting Information or are available from the corresponding author upon request.
