# Supplementary material for: Altered cytoskeletal integrity underlies impaired platelet shape change and defective thrombus formation in ETV6‐related thrombocytopenia
Source: Br J Haematol. 2026 Apr 28;208(6):1965–79. doi: 10.1111/bjh.70495 (PMC13267443; doi:10.1111/bjh.70495)
Supplement: Supplementary file 1 — Data S1. [file BJH-208-1965-s002.pdf]

SUPPLEMENTARY INFORMATION

Title: Altered cytoskeletal integrity underlies impaired platelet shape change and defective thrombus formation in ETV6-related thrombocytopenia

Authors: Ivan P. Tesakov[1,2], Julia-Jessica D. Korobkin[3], Daria V. Fedorova[2], Sofia V. Galkina[2,3], Maria M. Golomysova[3], Sergei I. Obydennyi[2,3], Anastasia A. Ignatova[2,3], Ekaterina-Iva A. Adamanskaya[2,3], Eugenia V. Yushkova[2,3], Anna V. Pavlova[2], Pavel A. Zharkov[2], Igor I. Kireev[4], Galina A. Novichkova[2], Viktor G. Zgoda[5], Mikhail A. Panteleev[2,3,6], Anastasia N. Sveshnikova[2,3,6]

Author affiliations: [1]Department of Oncology, Hematology, Clinical Immunology, and Rheumatology, University Hospital Tuebingen; Tuebingen, Germany; [2]Dmitry Rogachev National Medical Research Center of Pediatric Hematology, Oncology and Immunology; Moscow, Russia; [3]Center for Theoretical Problems of Physico-Chemical Pharmacology, Russian Academy of Sciences; Moscow, Russia; [4]A.N. Belozersky Institute of Physico-Chemical Biology, Lomonosov Moscow State University; Moscow, Russia; [5]Institute of Biomedical Chemistry, Moscow, Russia; [6]Lomonosov Moscow State University; Moscow, Russia

Corresponding author: Ivan P. Tesakov

SUPPLEMENTARY METHODS

Reagents

Tyrode's albumin buffer contained 137 mM NaCl, 2.7 mM KCl, 12 mM $NaHCO_3$, 0.36 mM NaH_2PO_4, 2 mM $CaCl_2$ (for Ca^{2+}-containing buffer), 1 mM $MgCl_2$, 20 mM HEPES, 0.36% bovine serum albumin (BSA), and 5.5 mM D-glucose. All salts were from Sigma-Aldrich (St. Louis, MO). Other reagents included: Fura-Red AM (Thermo Fisher Scientific, Waltham, MA); CalBryte-590 AM (AAT Bioquest, Sunnyvale, CA); ADP, EGTA, HEPES, BSA, apyrase grade VII, SFLLRN, AYGPKF, mepacrine (Sigma-Aldrich); human thrombin (Hematologic Technologies, Essex Junction, VT); collagen and ristocetin (Renam, Moscow, Russia); adrenaline (Moscow Endocrine, Moscow, Russia); cysteine-linked collagen-related peptide (CRP, CambCol Laboratories, Cambridge, UK); non-fibrillar human collagen type I (IMTEK, Moscow, Russia); CD62p-Alexa647, CD42b-PE, CD61-Alexa647, PAC1-FITC, and Annexin V-Alexa647 (Sony Biotechnology, San Jose, CA); and VM64 antibodies against PECAM-1/CD31 (RRID:AB_782149), a kind gift from Prof. A. V. Mazurov. All other reagents were from Sigma-Aldrich, unless otherwise indicated.

Molecular genetics

ETV6 variants were detected using targeted NGS panels (MiSeq/NextSeq, Illumina, USA) and confirmed by Sanger sequencing. Variant pathogenicity was assessed according to American College of Medical Genetics and Genomics (ACMG) guidelines[1].

Light transmission aggregometry

Aggregometry studies were performed as described[2]. Aggregation was assessed using a laser aggregometer (ALAT-2, Biola, Russia) in response to collagen (2 mg/mL), PAR1-activating peptide (PAR1-AP) SFLLRN (32 µM), adrenaline (5 µM), ADP (5 µM), and ristocetin (15 mg/mL).

Thromboelastography

Thromboelastography (TEG) was performed using a TEG 5000 Hemostasis Analyzer System (Haemonetics Corporation, Braintree, MA, USA). Native citrated whole blood samples were analyzed 10–40 min after collection. For each measurement, 340 µl of citrated blood was recalcified immediately prior to analysis with 20 µl of 0.2 M $CaCl_2$.

Single-platelet calcium imaging by TIRF microscopy

Single-platelet calcium responses were analyzed using total internal reflection fluorescence (TIRF) microscopy essentially as described previously[3]. Briefly, hirudin-anticoagulated whole blood was loaded with a calcium-sensitive dye CalBryte™ 590 AM (AAT Bioquest, USA) and perfused through parallel-plate flow chambers coated with either anti-CD31 (VM64) or collagen, after which platelets were allowed to adhere, washed, and imaged under baseline conditions or following stimulation with 10 µM ADP or 10 µM ADP plus 5 nM thrombin. Five-minute fluorescence traces were recorded for individual surface-immobilized platelets and classified into four predefined response groups (I–IV) according to their temporal patterns: Group I, fewer than five solitary spikes per 60 seconds; Group II, multiple stochastic spikes without clustering; Group III, frequent spikes merging into clusters without return to baseline; and Group IV, development of a sustained high calcium level during the recording. For each individual and condition, the percentage of platelets in each group was calculated and used for statistical comparison between ETV6-RT patients and healthy controls.

Transmission electron microscopy

Whole blood collected in sodium citrate tubes was left standing vertically for 1–2 hours to allow erythrocyte sedimentation and obtain platelet-rich plasma from the upper layer. Up to 500 µL of PRP was fixed in 1.25% glutaraldehyde solution in PBS. Standard sample preparation procedure included post-fixation with osmium tetroxide, graded acetone dehydration,

embedding in Epon 812 resin (Sigma-Aldrich, USA), block polymerization, ultramicrotomy (Leica UC7, Leica Microsystems, Germany), and double staining of sections with lead citrate and uranyl acetate. Transmission electron microscopy (TEM) imaging was performed using a JEOL JEM-1400 microscope (JEOL, Japan). Morphometric analysis of the resulting micrographs was performed according to a previously published method[4]. The control group included 20 healthy individuals from a cohort described previously[4].

Proteomics studies

Platelet isolation

Platelets were purified by triple centrifugation as described previously[5]. Blood was collected into sodium citrate (3.8% v/v) and platelet-rich plasma (PRP) was obtained by centrifugation at 100 × g for 8 minutes. PRP was supplemented with sodium citrate (27 mM) and centrifuged at 400 × g for 5 minutes. Platelets were resuspended in Tyrode's buffer, centrifuged again at 400 × g for 5 minutes, and resuspended in Tyrode's buffer to remove plasma traces. Purity of platelet suspensions prepared by this sequential centrifugation method has been reported previously[6]. For storage, platelets were pelleted at 2000 × g for 15 minutes and frozen in liquid nitrogen.

Protein extraction and digestion

Platelet pellets were lysed in 50 µL buffer containing 3% sodium deoxycholate and 100 mM Tris-HCl (pH 7.4). Samples were incubated on ice for 30 minutes and sonicated for 5 minutes. After centrifugation at 14,000 × g for 10 minutes at 4°C, the supernatant was collected and protein concentration measured with the BCA assay (Pierce, Thermo Scientific, USA). For reduction/alkylation, 50 µg protein was incubated with 4 mM TCEP and 6.2 mM CAA at 80°C for 30 minutes. Samples were digested overnight at 37°C with trypsin in 50 mM TEAB buffer (enzyme-to-protein ratio 1:50). Digestion was stopped with 5 µL formic acid, centrifuged at 16,000 × g for 10 minutes, and supernatants were vacuum dried and reconstituted in 20 µL 0.1% formic acid.

LC–MS/MS analysis

Peptides were separated on an Ultimate 3000 RSLCnano system (Thermo Scientific, USA) and analyzed on a Q-Exactive HFX mass spectrometer (Thermo Scientific, USA) with a 90-minute gradient.

Protein identification and quantification

MS/MS spectra were searched with MaxQuant v1.6.3.4[7] using the Andromeda algorithm[8] against the UniProt human proteome (UP000005640). Three replicate profiles were obtained per sample. Proteins were considered reliably identified if ≥2 unique peptides were detected and if they were previously reported in the platelet proteome. Label-free quantification (LFQ) was applied, and LFQ values correlated with reported copy numbers per platelet

Bioinformatics and statistics

Statistical analysis (Mann–Whitney test with FDR correction), LFQ histograms, and Venn diagrams were carried out in Python (Jupyter 7.3.2). Clustering and protein–protein interaction networks were generated with STRING v12.0.

Platelet spreading and F-actin staining

Parallel-plate flow chambers were assembled using silanized glass coverslips as previously described[3]. Chambers were coated with human fibrinogen (100 µg/mL in Tyrode's buffer without BSA or Ca^{2+}) for 50 min at room temperature, blocked with 5% BSA in Tyrode's buffer for 15 min, and washed with standard Tyrode's buffer. Chambers were equilibrated with Tyrode's buffer with Ca^{2+} prior to blood perfusion.

Whole blood was perfused at 10–33 µL/min for 2–5 min to form a platelet monolayer without thrombi, then washed with Ca^{2+}-containing Tyrode's buffer (5–10 min) to remove erythrocytes

and non-adherent platelets. Adherent platelets were further fixed by perfusion with 2% formalin (5 min) followed by static incubation (10 min), permeabilized with 0.1% Triton X-100 in PBS (5 min), and washed 3× with PBS (5 min/wash). Nonspecific binding was blocked with 3% BSA in PBS (1 h), followed by staining with Phalloidin-647 (1:100) and Hoechst 33342 (1:200) in 1% BSA/PBS. Chambers were washed 3× with 0.05% Triton X-100 in PBS (5 min/wash) and stored in 50% glycerol/150 mM NaCl/5 mM HEPES.

Platelet monolayers were imaged by fluorescence microscopy. Single platelets were segmented using ilastik pixel classifier (ilastik.org). Circularity was calculated as $4\pi S/P^2$, where S = platelet area and P = perimeter[9,10].

Immunofluorescence staining of peripheral blood smears

Immunofluorescence microscopy on standard air-dried peripheral blood smears was performed essentially as previously described[11,12]. Venous blood was collected into tubes containing 3.2–3.8% sodium citrate, and smears were prepared within 1 h and air-dried for at least 30 min at room temperature. Smears were fixed in cold acetone (−20°C, 2 min), air-dried completely, and either stained immediately or stored at −20°C for up to 2 weeks.

Before staining, a hydrophobic barrier was drawn around the area of interest. Slides were blocked with 10% goat serum in PBS (pH 7.2) for 30 min at room temperature in a humidified chamber. After removal of excess serum, primary antibodies diluted in PBS were applied and incubated for 1 h at room temperature: mouse anti-LAMP1 (clone H5G11, Santa Cruz; 1:20), mouse anti-β1-tubulin (clone TUB 2.1, Sigma-Aldrich; 1:100), and rabbit polyclonal anti–non-muscle myosin IIA (Sigma-Aldrich; 1:10,000). Slides were then washed three times in PBS (5 min each).

Fluorescent secondary antibodies (Alexa Fluor 488–conjugated goat anti-mouse IgG and Alexa Fluor 568–conjugated goat anti-rabbit IgG, BioLegend; each 1:400 in PBS) were applied for 1 h at room temperature in the dark, followed by three PBS washes (5 min each). Smears were mounted with coverslips using aqueous mounting medium.

Images were acquired on a Nikon Eclipse Ni-E fluorescence microscope with a 60× oil-immersion objective and a Nikon DS-Ri2 camera. Quantitative analysis was performed in Fiji; 50 platelets per sample were evaluated. All images from a given experiment were processed with identical settings, and expression pattern and subcellular localization of LAMP1, β1-tubulin, and non-muscle myosin IIA were assessed relative to healthy controls stained in parallel.

NETosis assay

NETosis was evaluated in leukocyte-rich plasma smears from EDTA-anticoagulated blood, prepared and stained essentially as previously described[13]. Neutrophils were identified by myeloperoxidase (MPO) and human neutrophil elastase (hNE) double-positive immunostaining with Hoechst 33342 DNA counterstain, and NETotic cells were quantified microscopically as those displaying extracellular MPO/hNE/DNA-positive structures.

SUPPLEMENTARY FIGURES

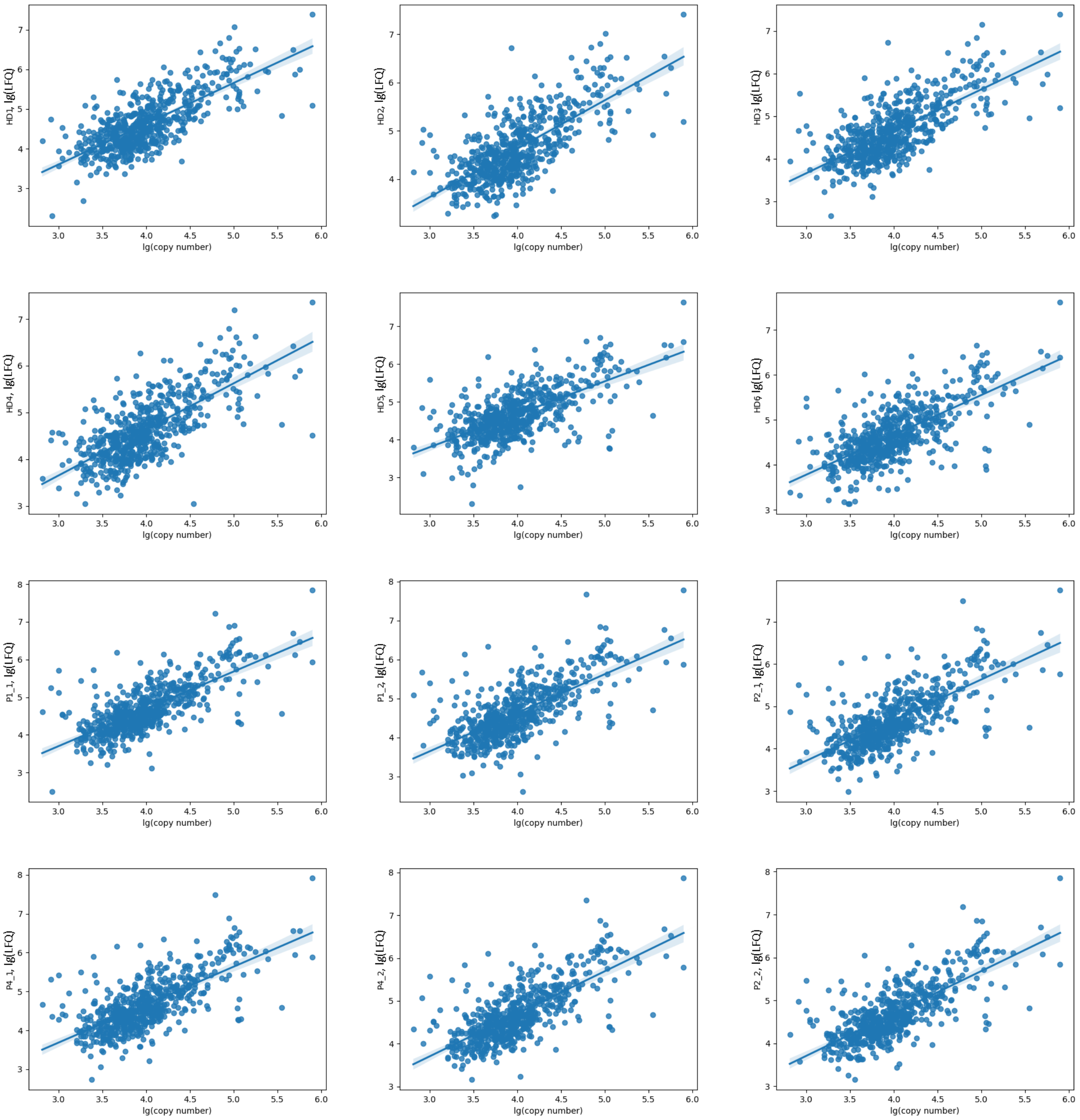

Supplementary Figure S1. Correlation of LFQ data with platelet copy numbers. Each panel depicts the subject named on the y-axis. Each dot corresponds to the base-10 logarithm of the mean LFQ value across technical replicates versus the base-10 logarithm of the platelet copy number for the same protein as previously reported[14].

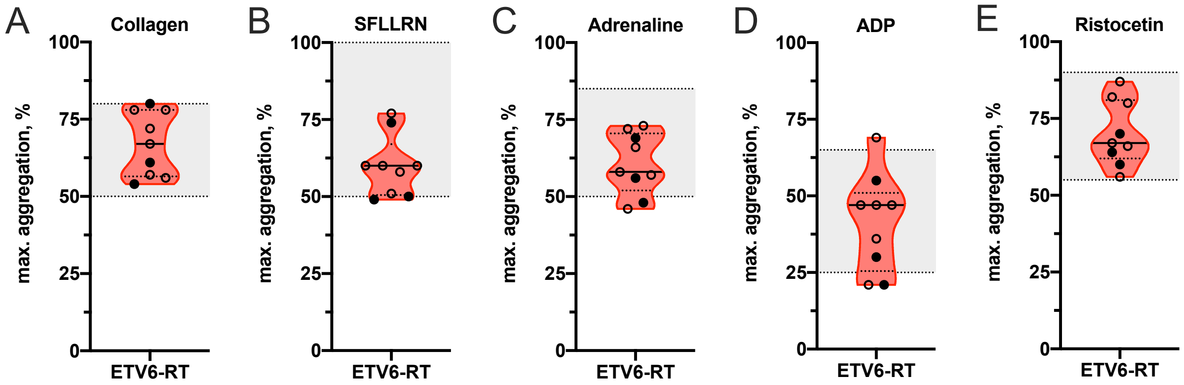

Supplementary Figure S2. Light transmission aggregometry in patients with ETV6-related thrombocytopenia upon stimulation with different agonists: (**A**) 2 mg/mL collagen, (**B**) 32 µM PAR1-activating peptide SFLLRN, (**C**) 5 µM adrenaline, (**D**) 5 µM adenosine diphosphate, and (**E**) 15 mg/mL ristocetin. Data are presented as violin plots showing the distribution of maximal platelet aggregation values, with width representing data density. Individual measurements are shown as circles, and data points from individuals carrying novel *ETV6* variants (Patients 3, 4.1, and 4.2) are highlighted as black circles; solid horizontal lines indicate the median, and dotted horizontal lines indicate the 25th and 75th percentiles. Gray shaded areas indicate normal reference ranges for each agonist (5th–95th percentile range of maximal aggregation obtained from 10 healthy individuals). PAR1-AP – protease-activated receptor 1–activating peptide; ADP – adenosine diphosphate.

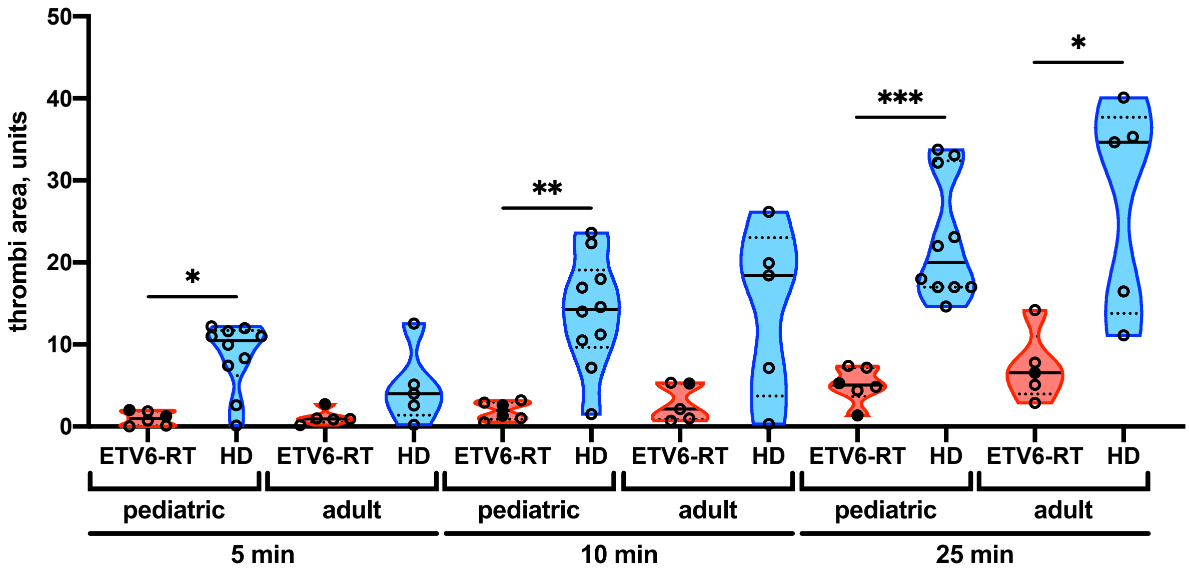

Supplementary Figure S3. Collagen-induced platelet thrombus formation in ETV6-related thrombocytopenia. Platelet thrombus area was measured 5, 10, and 25 minutes after the start of perfusion in pediatric and adult patients with ETV6-RT and age-matched healthy donors (HD). Data are presented as violin plots showing the distribution of values, with width representing data density. Individual measurements are shown as circles, with ETV6-RT patients in red and HD in blue, and data points from individuals carrying novel *ETV6* variants (Patients 3, 4.1, and 4.2) are highlighted as black circles; solid horizontal lines indicate the median, and dotted horizontal lines indicate the 25th and 75th percentiles. Statistical comparisons were performed using the Mann–Whitney U test. *$p < 0.05$; **$p < 0.01$; ***$p < 0.001$; absence of a symbol indicates no statistically significant difference.

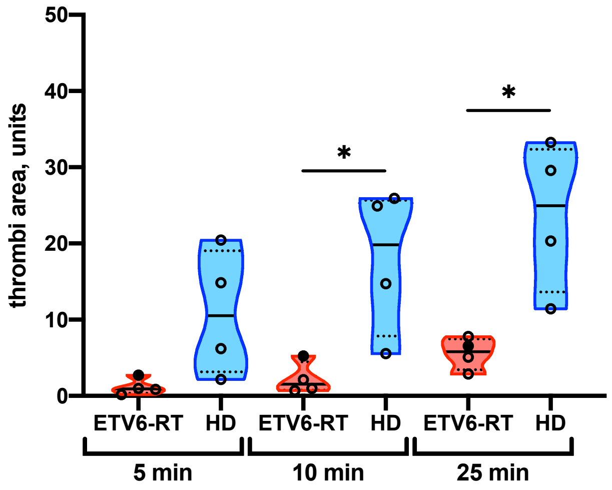

Supplementary Figure S4. Collagen-induced platelet thrombus formation in ETV6-related thrombocytopenia at matched platelet counts. Whole blood from adult ETV6-RT patients with platelet counts ≥100 × 10^9/L on the day of the experiment (Patients 1.1, 2.1, 4.1, and 5.1) and from adult healthy donors (HD) was perfused over fibrillar collagen in parallel-plate flow chambers. For HD, platelet counts were adjusted to 100 × 10^9/L by dilution of whole blood with autologous platelet-poor plasma as described[15]. Platelet thrombus area was measured 5, 10, and 25 minutes after the start of perfusion. Data are presented as violin plots showing the distribution of values, with width representing data density. Individual measurements are shown as circles, with ETV6-RT patients in red and HD in blue; data points from the individual carrying a novel *ETV6* variant (Patient 4.1) are highlighted as black circles. Solid horizontal lines indicate the median, and dotted horizontal lines indicate the 25th and 75th percentiles. Statistical comparisons were performed using the Mann–Whitney U test. *$p < 0.05$; absence of a symbol indicates no statistically significant difference.

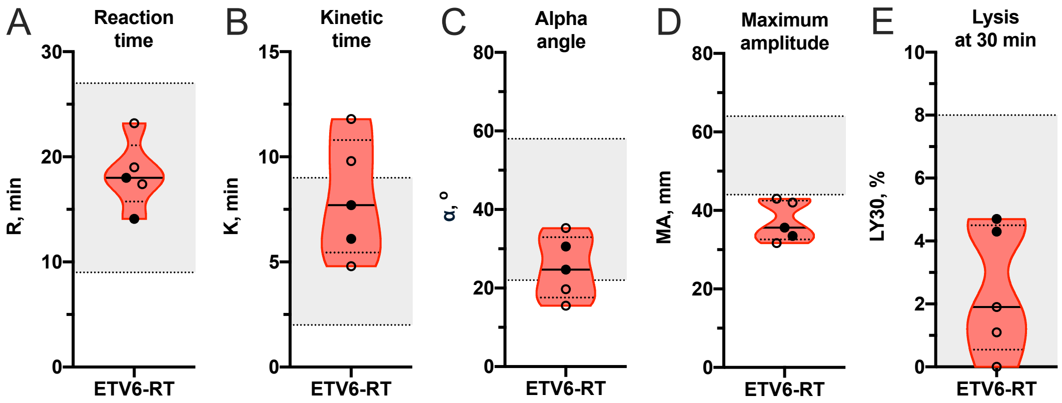

Supplementary Figure S5. Thromboelastography in patients with ETV6-related thrombocytopenia. Representative thromboelastography parameters obtained in ETV6-RT patients (Patients 1.1, 3, 4.2, 5.1, and 5.2): (**A**) reaction time (R), (**B**) kinetic time (K), (**C**) α-angle, (**D**) maximum clot amplitude (MA), and (**E**) fibrinolysis at 30 min (LY30). Data are presented as violin plots showing the distribution of values, with width representing data density. Individual measurements are shown as circles, and data points from individuals carrying novel *ETV6* variants (Patients 3 and 4.2) are highlighted as black circles; solid horizontal lines indicate the median, and dotted horizontal lines indicate the 25th and 75th percentiles. Gray shaded areas indicate reference ranges provided by the assay manufacturer.

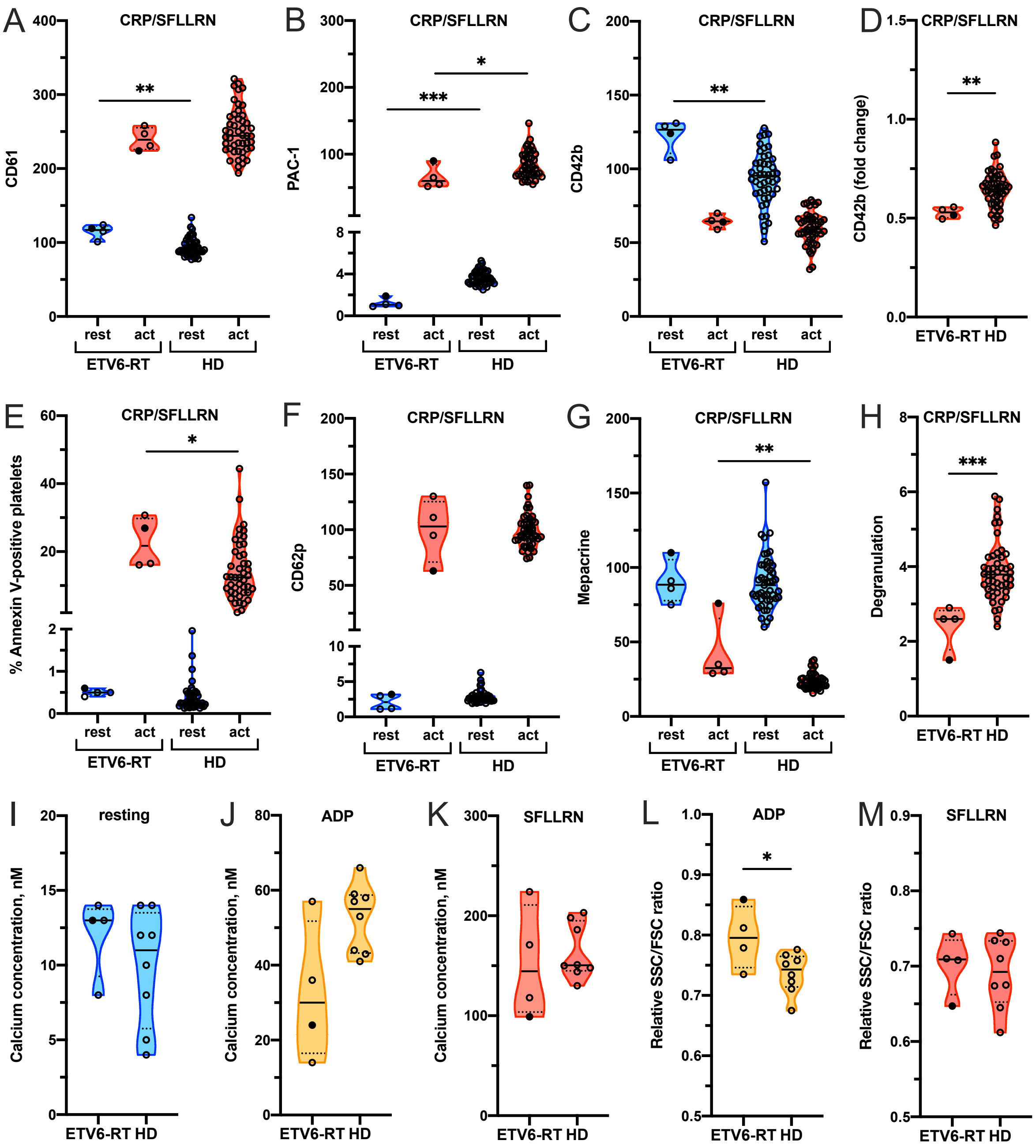

Supplementary Figure S6. Flow cytometry analysis of platelet function in adult patients with ETV6-related thrombocytopenia. Platelet functional parameters were assessed in adult ETV6-RT patients and healthy donors (HD) under resting conditions (**A–H**, 'rest'; **I**), after strong dual-agonist stimulation with 10 µg/mL collagen-related peptide (CRP) plus 12.5 µM PAR1-activating peptide (SFLLRN) (**A–H**, 'act'), and after stimulation with 2 µM ADP (**J,L**) or 5 µM SFLLRN (**K,M**). (**A**) Surface expression of GPIIIa (CD61) in resting platelets and after dual-agonist stimulation. (**B**) Expression of active GPIIb/IIIa (PAC-1 binding) in resting platelets and after dual-agonist stimulation. (**C**) Surface expression of GPIb (CD42b) in resting platelets and after dual-agonist stimulation. (**D**) Activation-induced CD42b fold change (ratio of CD42b MFI after dual-agonist stimulation to resting MFI), reflecting GPIb loss from the platelet surface. (**E**) Percentage of Annexin V–positive (procoagulant) platelets in resting conditions and after dual-agonist stimulation. (**F**) P-selectin (CD62p) expression in resting platelets and after dual-agonist stimulation. (**G**) Dense-granule content in resting platelets and after dual-agonist stimulation, measured as mepacrine fluorescence intensity. (**H**) Dense-granule release after dual-agonist stimulation, expressed as the activation-induced change in mepacrine signal (degranulation index; ratio of resting to post-stimulation fluorescence). (**I**) Cytosolic calcium

concentration in resting platelets. (**J,K**) Calcium mobilization following stimulation with ADP (**J**) or SFLLRN (**K**). (**L,M**) Platelet shape change after stimulation with ADP (**L**) or SFLLRN (**M**), expressed as the relative change in SSC/FSC ratio. Data are presented as violin plots showing the distribution of values, with width representing data density. Individual measurements are shown as circles, and data points from the individual carrying a novel *ETV6* variant (Patient 4.1) are highlighted as black circles; solid horizontal lines indicate the median, and dotted horizontal lines indicate the 25th and 75th percentiles. Statistical comparisons were performed using the Mann–Whitney U test. *$p < 0.05$; **$p < 0.01$; ***$p < 0.001$; absence of a symbol indicates no statistically significant difference.

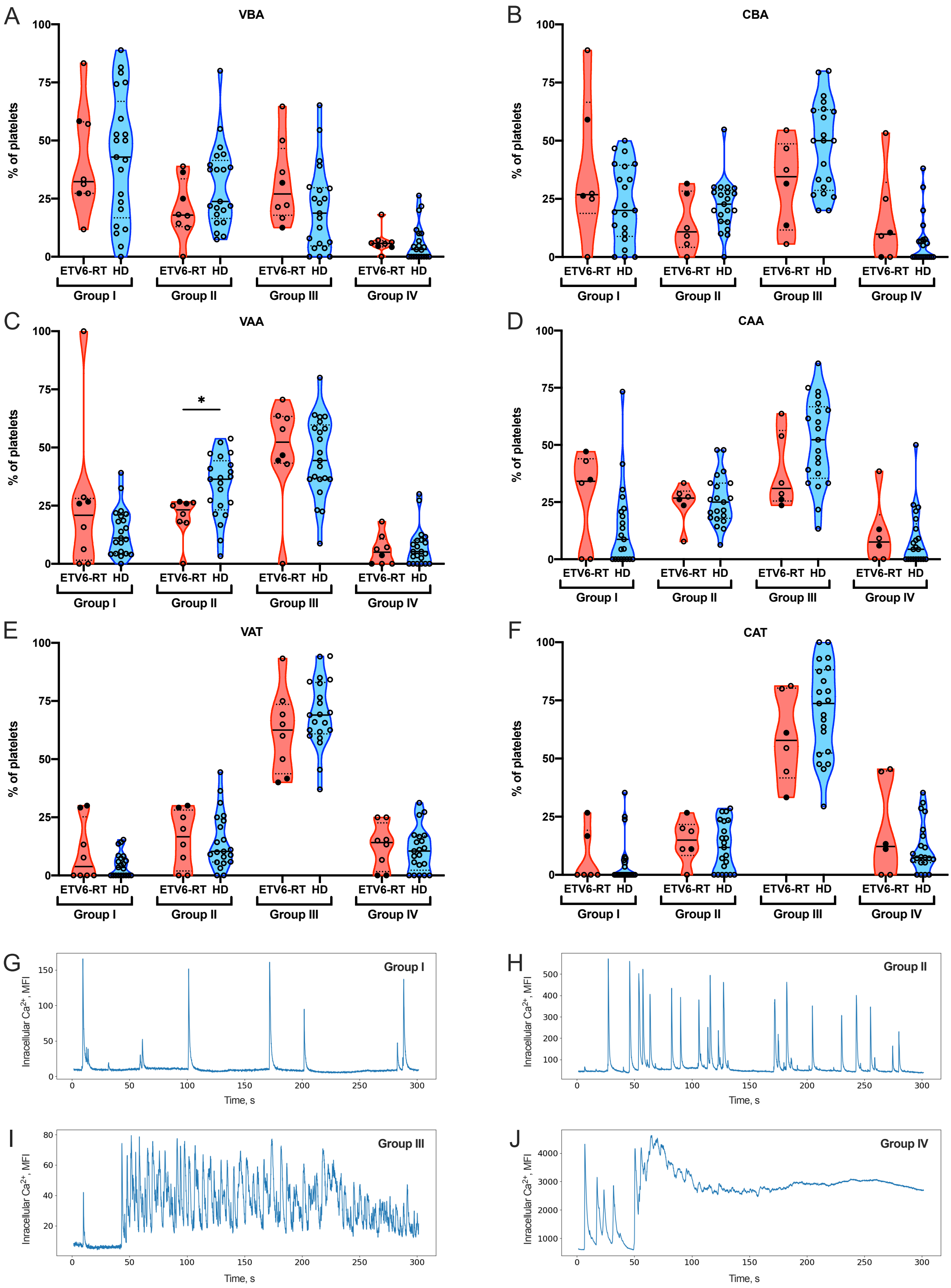

Supplementary Figure S7. Heterogeneity of single-platelet calcium responses in ETV6-related thrombocytopenia. Platelets were loaded with a calcium-sensitive dye and allowed to adhere to VM64- or collagen-coated surfaces in parallel-plate flow chambers, and single-platelet calcium dynamics were recorded by total internal reflection fluorescence (TIRF) microscopy as described previously[3]. Calcium responses were classified into four activation groups (I–IV) based on their temporal patterns. (**A**–**F**) Bar plots show the percentage of platelets in each group under six experimental conditions: (**A**) VBA (VM64, baseline), (**B**) CBA (collagen, baseline), (**C**) VAA (VM64 + 10 µM ADP), (**D**) CAA (collagen + 10 µM ADP), (**E**) VAT (VM64 + 10 µM ADP + 5 nM thrombin), and (**F**) CAT (collagen + 10 µM ADP + 5 nM thrombin), for ETV6-RT

patients (red) and healthy donors (blue). Data are presented as violin plots showing the distribution of values, with width representing data density. Individual measurements are shown as circles; data points from individuals carrying novel *ETV6* variants (Patients 4.1 and 4.2) are highlighted as black circles; solid horizontal lines indicate the median, and dotted horizontal lines indicate the 25th and 75th percentiles. Statistical comparisons were performed using the Mann–Whitney U test. *$p < 0.05$; absence of a symbol indicates no statistically significant difference. (**G–J**) Representative cytosolic calcium traces for the four response groups recorded in Patient 4.2: (**G**) Group I, with fewer than five solitary calcium spikes per 60 seconds; (**H**) Group II, showing multiple stochastic spikes that do not form clusters; (**I**) Group III, where frequent spikes merge into clusters without returning to baseline; and (**J**) Group IV, characterized by a sustained high cytosolic calcium level at some time point.

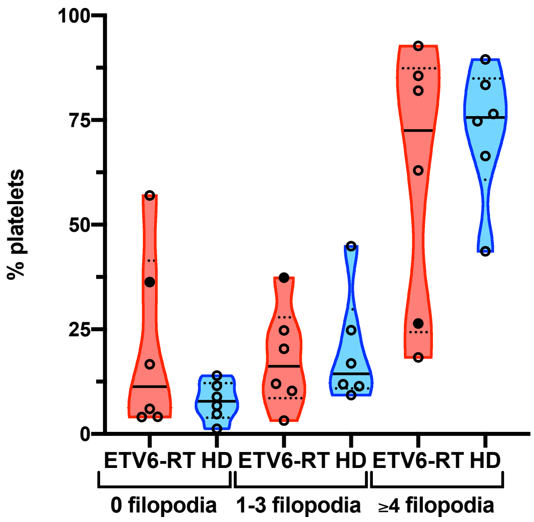

Supplementary Figure S8. Filopodia formation during platelet spreading in ETV6-related thrombocytopenia. Quantitative analysis of filopodia in spread platelets from six ETV6-RT patients (Patients 1.1, 1.2, 4.2, 6.1, 6.2, 6.3) and six healthy donors (91–276 platelets evaluated per individual). Platelets were classified into three categories according to filopodia count: 0 filopodia, 1–3 filopodia, or ≥4 filopodia. Data are presented as violin plots showing the percentage of platelets in each category, with width representing data density. Individual measurements are shown as circles (ETV6-RT patients in red, healthy donors (HD) in blue); data points from the patient carrying a novel *ETV6* variant (Patient 4.2) are highlighted as black circles. Solid horizontal lines indicate the median; dotted horizontal lines indicate the 25th and 75th percentiles. No significant differences were observed between groups (Mann–Whitney U test).

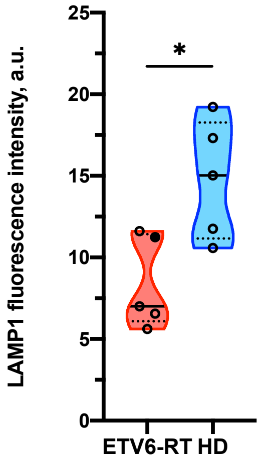

Supplementary Figure S9. Platelet LAMP1 expression in ETV6-related thrombocytopenia. Quantitative analysis of LAMP1 expression (mean gray value) in platelets from five ETV6-RT patients (Patients 1.1, 4.2, 6.1, 6.2, 6.3) and five healthy donors (50 platelets evaluated per individual). Data are presented as violin plots showing the distribution of mean gray values, with width representing data density. Individual measurements are shown as circles (ETV6-RT patients in red, healthy donors (HD) in blue); data points from the patient carrying a novel *ETV6* variant (Patient 4.2) are highlighted as black circles. Solid horizontal lines indicate the median; dotted horizontal lines indicate the 25th and 75th percentiles. Statistical comparisons by Mann–Whitney U test. *$p < 0.05$.

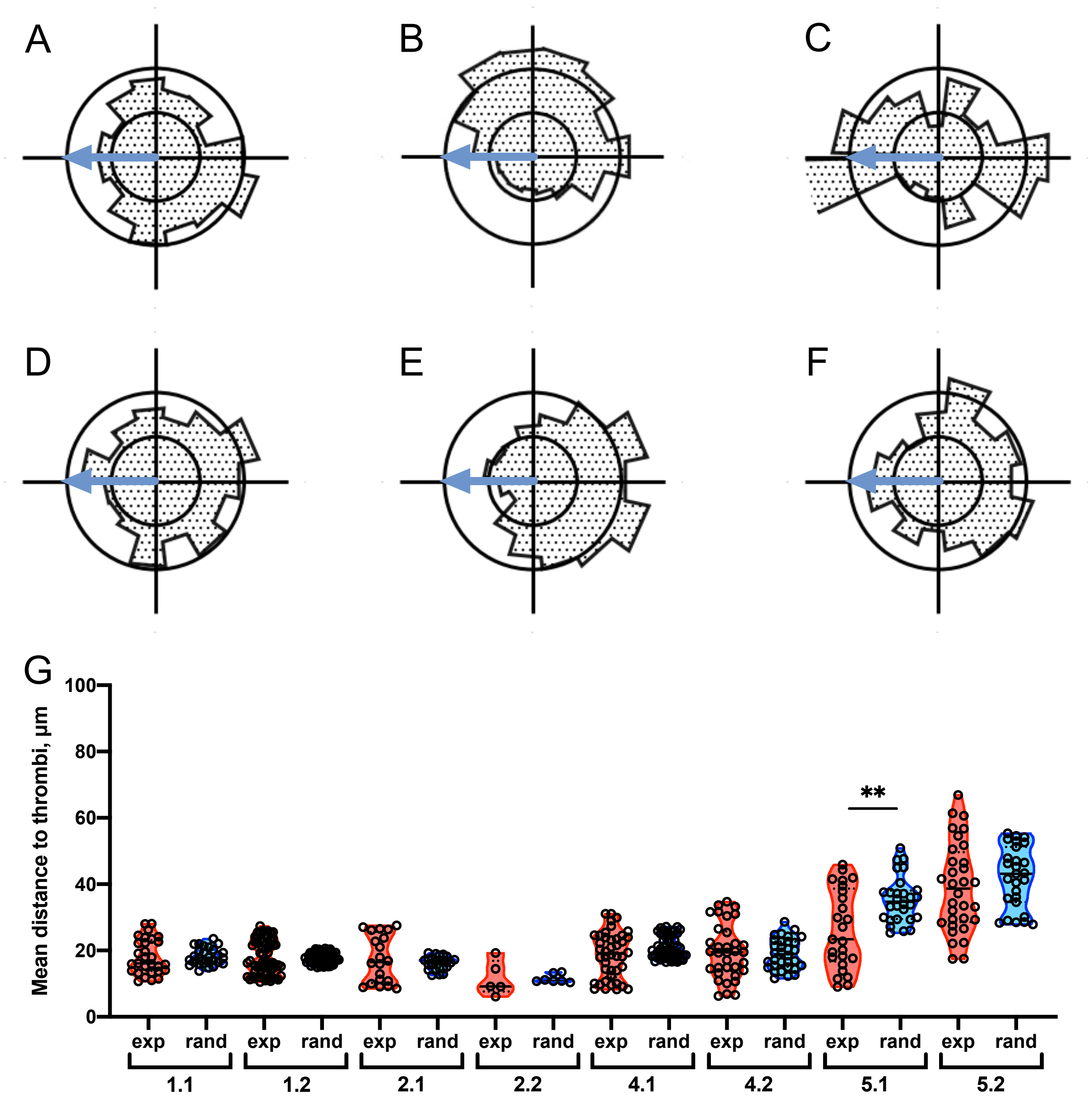

Supplementary Figure S10. Granulocyte migration within developing thrombi in ETV6-related thrombocytopenia. (**A–F**) Polar plots of granulocyte movement direction in a healthy donor (**A**) and ETV6-RT patients: Patient 1.1 (**B**), Patient 2.2 (**C**), Patient 4.1 (**D**), Patient 5.1 (**E**), Patient 5.2 (**F**). The blue arrow indicates flow direction. (**G**) The distance from the nearest thrombus edge to the center of each granulocyte compared with the distance from the thrombus border to a randomly placed, neutrophil-sized circle. Data are presented as violin plots showing the distribution of mean distance to thrombi, with width representing data density. Individual measurements are shown as circles (experimental in red, randomly generated in blue). Solid horizontal lines indicate the median; dotted horizontal lines indicate the 25th and 75th percentiles. Statistical comparisons were performed using the Mann–Whitney U test. **p < 0.01; absence of a symbol indicates no statistically significant difference.

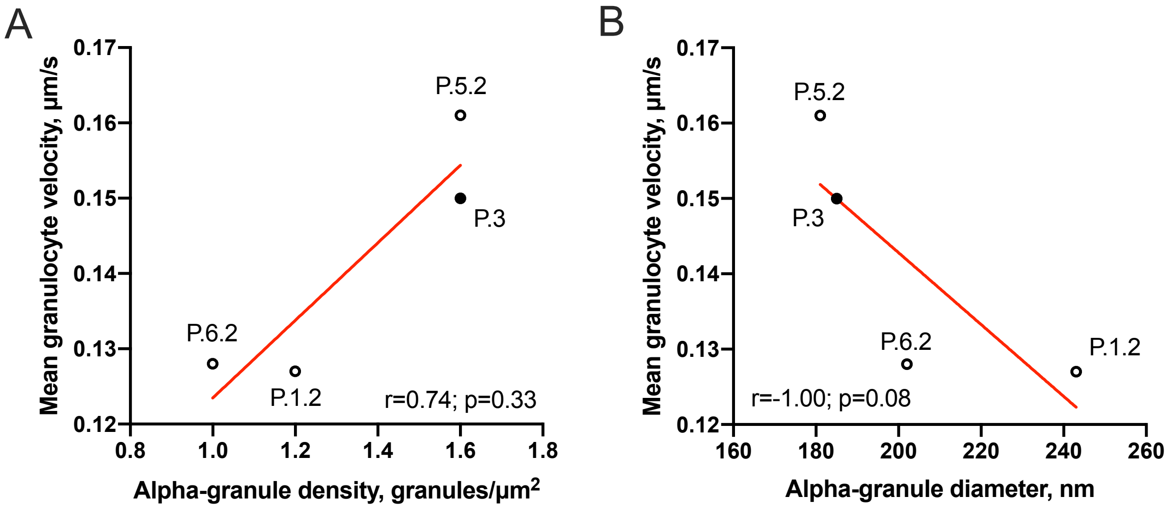

Supplementary Figure S11. Relationship between mean velocities of thrombus-associated granulocytes and alpha-granule characteristics in patients with ETV6-related thrombocytopenia. Mean granulocyte velocity is plotted against mean alpha-granule density (**A**) and mean alpha-granule diameter (**B**). Individual measurements are shown as circles, with data from the patient carrying the novel *ETV6* variant (Patient 3) highlighted in black. Red lines depict the linear regression trends. Spearman's rank coefficients (r) and corresponding p-values are reported to quantify the strength of association.

SUPPLEMENTARY TABLES

Supplementary Table S1. *In silico* predictions of the mutation effect on protein structure and conservation scores

Variant	Polyphen-2 HDIV/HVAR	SIFT	PROVEAN	CADD	GERP RS	PhyloP100way
c.1148A>G p.H383R	1.000/1.000	0.201	-6.19	29.2	5.63	9.343
c.1105C>T p.R369W	1.000/1.000	0.000	-6.37	32	5.63	5.952
c.1172A>G p.Y391C	1.000/1.000	0.000	-7.54	32	4.95	9.291
c.1192C>G p.L398V	1.000/1.000	0.000	-2.37	26.4	4.04	4.045
c.641C>T p.P214L	0.848/0.121	0.008	-2.46	22.9	5.65	6.811

In silico prediction tools

The potential pathogenicity of identified *ETV6* variants was evaluated using several algorithms:

- **PolyPhen-2** (http://genetics.bwh.harvard.edu/pph2/) predicts the impact of amino acid substitutions on protein stability and function using structural and evolutionary information. Scores range from 0.0 (benign) to 1.0 (damaging). Interpretation: 0.0–0.15 = benign, 0.15–0.85 = possibly damaging, 0.85–1.0 = probably damaging.
- **SIFT** (Sorting Intolerant From Tolerant; https://sift.bii.a-star.edu.sg/) evaluates nonsynonymous variants based on sequence homology. Scores range from 0 to 1, with values <0.05 considered damaging.
- **PROVEAN** (http://provean.jcvi.org/) predicts functional impact based on alignment-derived scores. Scores range from approximately –14 to +14. The default threshold is –2.5, below which variants are classified as deleterious.
- **CADD** (Combined Annotation Dependent Depletion; https://cadd.gs.washington.edu/) integrates multiple annotations to estimate deleteriousness. It provides a phred-like score; higher values indicate greater likelihood of pathogenicity. A score ≥20 is often used as a cutoff for potentially deleterious variants.

Conservation scores

- **GERP** (http://mendel.stanford.edu/SidowLab/downloads/gerp/) quantifies evolutionary constraint across 35 mammalian genomes. Scores range from –12.3 (least conserved) to +6.17 (most conserved), expressed as rejected substitutions (RS).
- **PhyloP100way** (UCSC Genome Browser: https://genome.ucsc.edu/) measures evolutionary conservation across 99 vertebrate genomes; higher scores indicate stronger conservation.

Supplementary Table S2. Characteristics of healthy individuals enrolled as controls for platelet proteomics studies

Donor	Age (years)	Gender	Hemostatic status#			
			D	T	B	F
G1.1	3	F	-	-	-	-
G1.5	2.5	M	-	-	-	-
G1.6	10	M	-	-	-	-
G2.3	12	F	-	-	-	-
G3.1	25	F	-	-	-	-
G3.3	27	F	-	-	-	-

#Characteristics of hemostatic status: D – history of coagulation/platelet disorders, T – anti-coagulant/ani-platelet therapy within 14 days of the study, B – history of profound or frequent nose-bleedings, skin or mucus hemorrhages, or surgical bleedings, F – history of coagulation/platelet disorders in close relatives.

Supplementary Table S3 (Excel file). STRING database annotations for the differentially expressed proteins between ETV6-RT patients and healthy donors. The proteins were considered differentially expressed if their amounts significantly (p-value <0.05) differed more than 2-fold.

REFERENCES

1. Richards S, Aziz N, Bale S, et al. Standards and guidelines for the interpretation of sequence variants: a joint consensus recommendation of the American College of Medical Genetics and Genomics and the Association for Molecular Pathology. *Genet Med*. 2015;17(5):405-424. doi:10.1038/gim.2015.30

2. Poletaev AV, Koltsova EM, Ignatova AA, et al. Alterations in the parameters of classic, global, and innovative assays of hemostasis caused by sample transportation via pneumatic tube system. *Thrombosis Research*. 2018;170:156-164. doi:10.1016/j.thromres.2018.08.024

3. Balabin FA, Galkina SV, Dzhamaliddinova R, et al. Heterogeneity of Single-platelet Calcium Responses to Activation. *Hamostaseologie*. Published online 30 January 2026:a-2773-1622. doi:10.1055/a-2773-1622

4. Kuznetsova AA, Kireev II, Obydennyi SI. A morphometric analysis of platelets using transmission electron microscopy. *Pediatric Hematology/Oncology and Immunopathology*. 2025;23(2):140-144. doi:10.24287/1726-1708-2024-23-2-140-144

5. Garzon Dasgupta AK, Martyanov AA, Ignatova AA, et al. Comparison of platelet proteomic profiles between children and adults reveals origins of functional differences. *Pediatr Res*. 2024;95(4):966-973. doi:10.1038/s41390-023-02865-y

6. Best MG, Sol N, Kooi I, et al. RNA-Seq of Tumor-Educated Platelets Enables Blood-Based Pan-Cancer, Multiclass, and Molecular Pathway Cancer Diagnostics. *Cancer Cell*. 2015;28(5):666-676. doi:10.1016/j.ccell.2015.09.018

7. Tyanova S, Temu T, Cox J. The MaxQuant computational platform for mass spectrometry-based shotgun proteomics. *Nat Protoc*. 2016;11(12):12. doi:10.1038/nprot.2016.136

8. Cox J, Neuhauser N, Michalski A, Scheltema RA, Olsen JV, Mann M. Andromeda: a peptide search engine integrated into the MaxQuant environment. *J Proteome Res*. 2011;10(4):1794-1805. doi:10.1021/pr101065j

9. Khan AO, Maclachlan A, Lowe GC, et al. High-throughput platelet spreading analysis: a tool for the diagnosis of platelet-based bleeding disorders. *Haematologica*. 2020;105(3):e124-e128. doi:10.3324/haematol.2019.225912

10. Kraus MJ, Neeb H, Strasser EF. Fractal and Euclidean descriptors of platelet shape. *Platelets*. 2014;25(7):488-498. doi:10.3109/09537104.2013.842639

11. Zaninetti C, Leinøe E, Lozano ML, et al. Validation of immunofluorescence analysis of blood smears in patients with inherited platelet disorders. *Journal of Thrombosis and Haemostasis*. 2023;21(4):1010-1019. doi:10.1016/j.jtha.2022.12.031

12. Yushkova EV, Podoplelova NA, Fedorova DV, et al. A single-center experience of using immunofluorescence staining of blood smears for the diagnosis of hereditary thrombocytopathies. *Pediatric Hematology/Oncology and Immunopathology*. 2023;22(3):43-47. doi:10.24287/1726-1708-2023-22-3-43-47

13. Adamanskaya EIA, Korobkin JJD, Pshonkin AV, et al. NETosis and Neutrophil Activity Quantification in Pediatric Patients with Essential Thrombocythemia. *IJMS*. 2025;26(24):11958. doi:10.3390/ijms262411958

14. Huang J, Swieringa F, Solari FA, et al. Assessment of a complete and classified platelet proteome from genome-wide transcripts of human platelets and megakaryocytes covering platelet functions. *Sci Rep*. 2021;11(1):12358. doi:10.1038/s41598-021-91661-x

15. Morozova DS, Martyanov AA, Obydennyi SI, et al. Ex vivo observation of granulocyte activity during thrombus formation. *BMC Biol*. 2022;20(1):32. doi:10.1186/s12915-022-01238-x
